# Cancer Incidence and Mortality Across 43 Cancer Registries in India

**DOI:** 10.1001/jamanetworkopen.2025.27805

**Published:** 2025-08-20

**Authors:** National Cancer Registry Programme Investigator Group, Prashant Mathur, Krishnan Sathishkumar, Priyanka Das, Stephen Santhappan, Jayasankar Sankarapillai, Anita Nath, Monesh Baburao Vishwakarma, Rajaraman Swaminathan, Sampath Pitchaimuthu, Sankaravamsam Venkata Suryanarayana Deo, Nalliah Manoharan, Vinay Deshmane, Shravani Koyande, Sudeep Gupta, Atul Budukh, Boraiah Thejaswini, CR Vijay, Aleyamma Mathew, Preethi Sara George, Satheesan Balasubramanian, Saina Sunilkumar, Shashank Pandya, Anand Shah, Jayanta Chakrabarti, Debasish Jatua, Rekha A. Nair, Arshad Manzoor Najmi, Shaqul Qamar Wani, Sadashivudu Gundeti, Gautam Majumdar, Shiromani Debbarma, B. Paul Thaliath, Radharani Ghosh, Umesh Mahantshetty, Dolorosa Fernandes, Satyajit Pradhan, Divya Khanna, Debabrata Barmon, Tashnin Rahman, Reeni Malik, Atul Shrivastava, Wallambok Langstieh, Vikas Jagtap, Eric Zomawia, Lalchhandama Chhakchhuak, Sushma Khuraijam, Rajesh Singh Laishram, Nandakumar Panse, Vijaya Dulange, Shah Alam Sheikh, Ajit Kumar Dey, Ashish Gulia, Vandita Pahwa, Smita Asthana, Shalini Singh, Vijay Kumar Bodal, Mohanvir Kaur, Anupama Gupta, Jarnail S. Thakur, Rajesh Dikshit, R. Ravi Kannan, Ritesh Tapkire, Suvarna Patil, Monika Sarade, Adity Sharma, Zarika Ahmed, Pankaj Chaturvedi, Ravikant Singh, Ashok Tshering Sherpa, Priya D. Pradhan, Deepali Lokhande, Sushama Saoba, Vinotsole Khamo, K. Shevo Hiese, Sopai Tawsik, Nobin Hage, Kaling Jerang

**Affiliations:** 1 Indian Council of Medical Research, National Centre for Disease Informatics and Research, Bengaluru, India; 2 Cancer Institute (WIA), Adyar Chennai, India; 3 Department of Surgical Oncology, Dr B. R. Ambedkar Institute Rotary Cancer Hospital, All India Institute of Medical Science, New Delhi, India; 4 Delhi Cancer Registry, Dr B. R. Ambedkar Institute Rotary Cancer Hospital, All India Institute of Medical Science, New Delhi, India; 5 P. D. Hinduja Hospital, Mumbai, India; 6 Breach Candy Hospital, Mumbai, India; 7 Asian Cancer Institute Mumbai, Mumbai, India; 8 Mumbai, Pune, Nagpur, and Aurangabad Cancer Registry, Mumbai, India; 9 Mumbai Cancer Registry, India; 10 Tata Memorial Centre, Constituent Institution of Homi Bhabha National Institute, Navi Mumbai, India; 11 Centre for Cancer Epidemiology, Tata Memorial Centre, Constituent Institution of Homi Bhabha National Institute, Navi Mumbai, India; 12 Rajiv Gandhi University of Health Sciences, Bengaluru, India; 13 Division of Cancer Epidemiology & Biostatistics, Regional Cancer Centre, Thiruvananthapuram, India; 14 Regional Cancer Centre, Thiruvananthapuram, India; 15 Malabar Cancer Centre, Post Graduate Institute of Oncology Sciences & Research, Thalassery, India; 16 The Gujarat Cancer & Research Institute, Ahmedabad, India; 17 Chittaranjan National Cancer Institute, Kolkata, India; 18 Radiation Oncology, Kashmir Institute of Medical Sciences, Srinagar, India; 19 Department of Medical Oncology, Nizam’s Institute of Medical Sciences, Hyderabad, Telangana, India; 20 Tripura University, Agartala, India; 21 Medical College, Imphal, India; 22 Regional Cancer Centre, Kamala Nehru Memorial Hospital, Prayagraj, India; 23 Homi Bhabha Cancer Hospital & Research Centre, Tata Memorial Centre, Visakhapatnam, India; 24 Homi Bhabha Cancer Hospital and Mahamana Pandit Madan Mohan Malaviya Cancer Centre, Varanasi, India; 25 Dr B. Borooah Cancer Institute, Guwahati, India; 26 Gandhi Medical College, Bhopal, India; 27 State Cancer Registry Programme, Madhya Pradesh, Bhopal, India; 28 Meghalaya AIDS Control Society, Shillong, India; 29 Department of Radiation Oncology, North Eastern Indira Gandhi Regional Institute of Health & Medical Sciences, Shillong; 30 Population-Based Cancer Registry, Mizoram, India; 31 NHM Mizoram, Mizoram, India; 32 HOD Pathology Department, Civil Hospital, Aizawl, India; 33 Population-Based Cancer Registry, Regional Institute of Medical Science, Imphal, India; 34 Barshi Rural, Population Based Cancer Registry, Barshi, India; 35 Silchar Medical College, Silchar, India; 36 Homi Bhabha Cancer Hospital & Research Centre, Tata Memorial Centre, New Chandigarh, India; 37 Homi Bhabha Cancer Hospital, Tata Memorial Centre, Sangrur, India; 38 Indian Council of Medical Research–National Institute of Cancer Prevention and Research, Noida, India; 39 Population Based Cancer Registry, Patiala, India; 40 Mahatma Gandhi Institute of Medical Sciences, Sevagram, India; 41 Graduate Institute of Medical Education and Research, Chandigarh, India; 42 The Tamil Nadu Dr MGR Medical University, Chennai, India; 43 B. K. L. Walawalkar Rural Medical College & Hospital, Taluka-Chiplun, India; 44 Centre for Cancer Epidemiology, Tata Memorial Centre, Navi Mumbai, India; 45 Assam Medical College & Hospital, Dibrugarh, India; 46 Advanced Centre for Treatment, Research and Education in Cancer, Tata Memorial Centre, Constituent Institution of Homi Bhabha National Institute, Navi Mumbai, India; 47 Homi Bhabha Cancer Hospital & Research Centre, Tata Memorial Centre, Muzaffarpur, India; 48 Sir Thutob Namgyal Memorial Hospital, Sochakgang Hospital, Gangtok, India; 49 Nagaland Institute of Science and Technology, Kohima, India; 50 Department of Health and Family Welfare, Government of Nagaland, Kohima, India; 51 Tomo Riba Institute of Health and Medical Sciences, Naharlagun, India; 52 Bakin Pertin General Hospital, Pasighat, East Siang District, Arunachal Pradesh, India

## Abstract

**Importance:**

Cancer is a significant global health concern, with India ranking second in Asia and third in the world in terms of cancer incidence. Regular monitoring and updates on cancer statistics are vital for assessing the impact and burden of the disease and the effectiveness of cancer control measures.

**Objective:**

To measure the recent patterns and trends in cancer incidence and mortality across 43 geographic regions in India from 2015 to 2019 and to provide estimates for 2024.

**Design, Setting, and Participants:**

This cross-sectional study used data from 43 population-based cancer registries across India, covering varying periods between January 1, 2015, and December 31, 2019. Population at-risk data were obtained from the Census of India, and findings were assessed by registry area. Data were analyzed from May 1 to December 20, 2024.

**Main Outcomes and Measures:**

Number of cases, crude rates, and age-adjusted rates (per 100 000 population) for cancer incidence and mortality, estimated average annual percent change (AAPC) from time trends, and estimated cancer cases in India for 2024.

**Results:**

Incidence of 708 223 cases with 206 457 deaths from 43 population-based cancer registries were included. The lifetime risk of developing cancer in India was 11.0%, while Mizoram in the Northeastern region reported lifetime risks of 21.1% in males and 18.9% in females. The district of Aizawl reported the highest age-adjusted incidence rate (AAIR) in both males (256.1; 95% CI, 245.2-267.0) and females (217.2; 95% CI, 207.6-226.7). The most common cancers were oral, lung, and prostate in males and breast, cervical, and ovarian in females. Among metropolitan cities (defined as an urban agglomeration with a population of over 1 million), Delhi had the highest overall cancer AAIR for males (146.7; 95% CI, 145.1-148.3), while Srinagar recorded the highest AAIR for lung cancer (39.5; 95% CI, 35.8-43.2). Oral cancer showed significant increases in 14 population-based cancer registries (PBCRs) among males and 4 PBCRs among females; Ahmedabad Urban had an increase of 4.7% (95% CI, 2.9% to 6.6%) in males and 6.9% (95% CI, 4.1% to 9.7%) in females. The estimated AAPC in AAIR (all sites) showed a significant increase over time in Kamrup Urban in males (3.3%; 95% CI, 2.3%-4.3%) and Thiruvananthapuram Taluk in females (3.4%; 95% CI, 3.1%-3.8%). The estimated cancer incidence for 2024 was 1 562 099 cases; estimated cancer mortality, 874 404 cases.

**Conclusions and Relevance:**

This cross-sectional study highlighted significant regional disparities in cancer incidence across India and the increasing cancer burden. The findings provide key insights for policymakers to enhance resource allocation and strengthen cancer control strategies nationwide.

## Key Points

**Question:**

What are the recent patterns and trends in cancer incidence and mortality in India?

**Findings:**

In this cross-sectional National Cancer Registry Programme study, highlighting regional disparities in cancer rates across India, the lifetime risk of developing cancer in India was 11.0%, while Mizoram in the Northeastern region reported lifetime risks of 21.1% in males and 18.9% in females.

**Meaning:**

These findings underscore a need to strengthen the ongoing efforts for cancer prevention and control measures to reduce the burden of cancer in India.

## Teaser

This cross-sectional study provides a comprehensive analysis of recent patterns in cancer incidence and mortality across 43 geographic regions in India

## Introduction

As one of the leading causes of mortality and morbidity, cancer is a significant worldwide health concern. Globally, cancer contributes to approximately 10 million deaths each year.^1^ In 2022, the Global Cancer Observatory (GCO) estimated the total number of cancer cases worldwide at approximately 20.0 million and projected these to increase to 32.6 million by 2045.^1^ The region of Southeast Asia is estimated to have a total of 2.4 million new cancer cases and 1.5 million cancer deaths.^1^ Cancer incidence and mortality in this region are estimated to increase to 4.0 million new cases and 2.7 million deaths by 2045. Concurrently, the GCO estimated that the incidence of cancer in India will increase to approximately 2.46 million cases by 2045.^1^ India ranks second in Asia and third in the world in terms of the number of cancer cases, and the likelihood of developing cancer during one’s lifetime is approximately 11.0%.^1^ Previous publications from the National Cancer Registry Programme (NCRP) estimated 1.46 million cancer cases in 2022, corresponding to a crude incidence rate of 100.4 per 100 000 population in India.^2^

Cancer registries are widely acknowledged as an essential component of national cancer control programs.^3^ In India, data on cancer have been systematically collected since 1981 through the NCRP of Indian Council Medical Research, which is operated from the National Centre for Disease Informatics and Research through the network of population-based cancer registries (PBCRs) and hospital-based cancer registries. The Sustainable Development Goal 3, Target 3.4, aims to reduce premature mortality from cancer and other noncommunicable diseases by one-third by 2030.^4^ PBCRs contribute to this objective by systematically collecting data on the newly diagnosed cancer cases and related mortality within a specific geographic area. They also monitor and assess the burden of cancer, which plays a significant role in the assessment of control of cancer within those defined populations.^5,6,7,8,9,10^

This study provides a comprehensive analysis of recent patterns and trends in cancer incidence and mortality across 43 geographic regions in India using data from the composite period of January 1, 2015, to December 31, 2019. Additionally, it provides estimated cancer incidence and mortality cases in India for the year 2024.

## Methods

This report presents cancer incidence and mortality from 43 PBCRs across India, of which 33 are operated under the National Centre for Disease Informatics and Research, 9 are managed by the Tata Memorial Centre (TMC), Mumbai, and 1 is managed under the Tamil Nadu Cancer Registry Programme, together constituting the NCRP. These PBCRs covering the varying periods between 2015 and 2019 represent approximately 18% of India’s population. Trend analysis was conducted for 23 populations from January 1, 2002, to December 31, 2019. Cancer registration in PBCRs in India involves a multistep process that requires continuous and systematic data collection by trained skilled registry personnel from various sources, including diagnostic laboratories, hospitals, and vital statistics departments using a standardized proforma.^11^ All neoplasms characterized by a behavior code 3 according to the *International Classification of Diseases for Oncology, 3rd Edition*, and the *International Statistical Classification of Diseases and Related Health Problems, Tenth Revision* (*ICD-10*) were classified as reportable and subsequently recorded in the cancer registry (eTable 1 in Supplement 1).^12^ The Institutional Ethics Committee of National Centre for Disease Informatics and Research approved the study. Waiver of consent was obtained as the study used anonymized registry data. The study complied with the guidelines of the Strengthening the Reporting of Observational Studies in Epidemiology (STROBE) for reporting observational study findings.

Data quality indices were computed for each PBCR to assess the reliability of the data (microscopic verification, ≥75%; death certificate–only verification, <10%; other and unspecified sites of cancer, <10%). NCRP uses in-house PBCR data management software (PBCRDM, version 2.1.1 [National Centre for Disease Informatics and Research]) for data collection, quality control, duplicate removal, and linking mortality to incidence (M:I ratio). The registries under TMC collected data using CanReg-5 software.^13^ Data errors were flagged and sent back to all the registries for clarification, then cross-checked with the data source and corrected accordingly. The data quality was maintained according to the standards of the International Association of Cancer Registries and the International Agency for Research on Cancer.^14,15^

### Estimation of Incidence and Mortality

Incidence of cancer cases in India for 2024 was estimated using trends in the cancer incidence rate and projected population.^2,5,16^ We analyzed trends from 2010 to 2019 (state- or region-specific PBCR) to estimate cancer incidence rate by anatomical site and sex. For anatomical sites where the annual percent change (APC) was statistically significant, cancer incidence rates were estimated to 2024. For sites where the APC was not statistically significant, the incidence rates from 2015 to 2019 were assumed to remain constant until 2024. Data on the population at risk by state and sex were obtained from the Census of India.^17^ The estimated incidence rates for 2024 were then applied to respective projected population figures for each state, and for the entire country. Mortality estimates for India were modeled as a function of cancer incidence estimates and M:I ratios, stratified by anatomical site and sex, based on the Mumbai PBCR.

### Statistical Analysis

Data were analyzed from May 1 to December 20, 2024. We analyzed cancer incidence and mortality using data from all PBCRs (708 223 cases and 206 457 deaths) across varying periods between 2015 and 2019. Cancer statistics are presented as the number of incident cases, crude incidence rate (CIR), and age-adjusted incidence rate (AAIR) per 100 000 population using the Indian standard population (ISP) and World standard population (WSP).^18,19^ Similarly, the number of mortality cases, crude mortality rate (CMR), age-adjusted mortality rate (AAMR) per 100 000 population, and the M:I ratio were derived. While the WSP allows for international and historical comparisons, the ISP was used as part of a sensitivity analysis to assess the association of India’s population age structure with incidence estimates. The use of the ISP, being more representative of the population of India, enables subnational comparison within the country. Therefore, simultaneous observation of crude rates (actual cancer burden) and age-adjusted rates (comparison across the population) using the WSP and the ISP provided a more comprehensive understanding of the cancer burden in the country.^19^ Leading sites of cancer are presented based on the relative proportion (percentage) of the total cancer of the PBCR. To evaluate the lifetime risk of developing cancer, cumulative risk (0-74 years of age) was calculated. In a PBCR covering multiple districts, the district with the highest incidence rates was analyzed and presented separately (eg, Aizawl subgroup of the Mizoram PBCR). A small proportion of missing age data (0.1%) was not imputed. The change in incidence rates resulting from the inclusion of 2020 data (reflecting the impact of COVID-19) is presented for 10 different populations in (eTable 2 in Supplement 1). A comparative analysis of AAIR for leading cancer sites (breast, cervix, prostate, oral, stomach, and lung) was performed across the PBCRs based on the WSP (eFigure 1 in Supplement 1).

Time trends in AAIR for the 23 populations (2002-2019) were analyzed for all sites and leading cancer sites using the Joinpoint Regression Program, version 5.0.2.^20^ The model estimates the APC for each segment defined by a trend change and calculates the average APC (AAPC) for the entire study period by fitting a regression line to the natural logarithm of the AAIRs, under the assumption of constant variance. The grid search method was used for model fitting, with the maximum number of Joinpoints set to 2. The optimal number of Joinpoints was determined through a permutation test. Statistical significance was assessed using 2-sided tests, with *P* < .05 considered statistically significant.^20^ The Census growth rate was used to project the population at risk for each PBCR.^21,22^

## Results

An incidence of 708 223 cases (51.1% female and 48.9% male) and mortality of 206 457 cases (45.0% female and 55.0% male) were reported from 43 PBCRs between 2015 and 2019. Table 1 presents the distribution of cancer cases across all sites with incidence rate and cumulative risk by sex in different regions in India between 2015 and 2019. The dataset includes 43 PBCRs representing 56 distinct populations. The highest AAIR (using the ISP) was found in Aizawl for both males (198.4; 95% CI, 190.1-206.7) and females (172.5; 95% CI, 165.0-179.9), with the lowest rates recorded in Osmanabad and Beed for males (30.4; 95% CI, 29.5-31.3) and in Dima Hasao for females (22.1; 95% CI, 16.6-27.5). The lifetime risk of developing cancer in India was 11.0%, while Mizoram in the Northeastern region reported lifetime risks of 21.1% in males and 18.9% in females.

**Table 1.** Cancer Cases for All Sites, Incidence Rates Per 100 000 Population, and Cumulative Risk by Sex for 43 PBCRs (2015-2019), India

SN and PBCR (reference year)	2011 Census population, %		Males				Females			
	Urban	Rural	Cases, No.	CIR per 100 000 population	AAIR using ISP (95% CI)	Cumulative risk of cancer, aged 0-74 y, %	Cases, No.	CIR per 100 000 population	AAIR using ISP (95% CI)	Cumulative risk of cancer, aged 0-74 y, %
1										
Kashmir Province, Jammu and Kashmir (2018-2019)	68.0	32.0	7113	92.3	103.5 (101.1-105.9)	15.5	5570	81.7	86.2 (83.9-88.4)	11.2
Pulwama, Jammu and Kashmir (2018-2019)	14.4	85.6	694	131.6	132.3 (122.3-142.3)	18.6	612	130.8	121.4 (111.5-131.1)	15.5
Srinagar, Jammu and Kashmir (2018-2019)	98.6	1.4	2033	155.8	134.2 (128.3-140.1)	19.9	1628	132.9	111.2 (105.8-116.7)	14.0
2: Delhi, UT (2015 to 2017)	100	0	34 303	116.5	117.5 (116.2-118.8)	15.8	30 479	115.7	107.1 (105.8-108.2)	14.2
3: Gautam Buddha Nagar, Uttar Pradesh (2016-2018)	59.1	40.9	2906	89.4	101.5 (97.8-105.2)	14.7	2696	96.7	105.2 (101.2-109.2)	14.2
4: Prayagraj, Uttar Pradesh (2017-2019)	25.0	75.0	5542	51.8	53.4 (52.0-54.8)	7.2	4824	49.2	49.5 (48.1-50.9)	6.4
5: Varanasi, Uttar Pradesh (2018-2019)	43.4	56.6	2619	62.2	59.8 (57.5-62.1)	8.1	2013	52.3	48.9 (46.7-51.0)	6.5
6: Muzaffarpur, Bihar (2018)^a^	38.1	61.9	383	36.4	35.8 (32.2-39.5)	4.6	368	38.8	39.1 (35.0-43.2)	4.9
7: Patiala district, Punjab (2015-2018)	40.3	59.7	2960	66.8	55.8 (53.8-57.9)	7.8	3387	84.9	66.0 (63.7-68.2)	8.8
8: Sangrur, Punjab (2017-2018)	31.2	68.8	1338	70.2	58.7 (55.5-61.9)	8.4	1369	81.3	65.9 (62.4-69.4)	8.7
9: Mansa, Punjab (2017-2018)	21.3	78.7	561	63.7	49.0 (44.8-53.2)	6.7	617	79.1	56.8 (52.2-61.4)	7.9
10: Chandigarh, Punjab (2017-2018)	97.3	2.7	968	72.8	73.6 (68.9-78.3)	10.2	990	88.9	81.4 (76.3-86.6)	11.8
11: SAS Nagar, Punjab (2017-2018)	54.8	45.2	917	69.0	67.3 (62.8-71.7)	9.8	1030	85.5	81.8 (76.8-86.9)	11.0
12: Bhopal, Madhya Pradesh (2016-2019)	100	0	4638	98.2	91.7 (89.1-94.4)	12.3	4304	97.3	89.2 (86.5-91.9)	11.8
13: Ahmedabad Urban, Gujarat (2015-2018)	100	0	13 472	95.9	83.7 (82.3-85.2)	11.4	10 553	83.0	67.9 (66.6-69.2)	9.1
14: Cachar district, Assam (2015-2019)	18.2	81.8	5283	106.6	107.4 (104.5-110.3)	15.3	4637	96.5	96.8 (94.0-99.6)	12.1
15										
Karimganj, Assam (2016-2018)^b^	10.5	89.5	2192	60.7	65.4 (62.7-68.2)	9.4	1539	44.0	48.7 (46.3-51.2)	6.6
Hailakandi, Assam (2016-2018)	7.3	92.7	702	61.5	66.6 (61.7-71.6)	9.5	443	40.4	45.4 (41.1-49.6)	5.9
Dima Hasao, Assam (2016-2018)	29.2	70.8	129	36.3	42.3 (34.8-49.7)	6.5	65.0	19.0	22.1 (16.6-27.5)	3.3
Karimganj district, Assam (2016-2018)	8.9	91.1	1274	60.2	64.3 (60.7-67.8)	9.3	946	45.9	50.2 (47.0-53.4)	6.9
16: Dibrugarh district, Assam (2015-2018)	18.4	81.6	2073	72.4	69.3 (66.2-72.3)	10.2	1909	68.1	63.3 (60.4-66.1)	8.5
17: Kamrup Urban, Assam (2015-2018)	100	0	5384	195.5	163.3 (158.9-167.8)	22.2	4395	159.8	139.3 (135.1-143.5)	18.3
18: Tripura state (2015-2018)	26.2	73.8	6049	74.7	68.9 (67.1-70.7)	10.3	4645	59.3	53.0 (51.5-54.6)	7.1
19: Sikkim state (2015-2018)	25.2	74.8	1054	76.3	72.3 (67.9-76.8)	10.1	994	80.1	79.73 (74.7-84.7)	10.5
20										
Mizoram state (2015-2019)	52.1	47.9	4501	143.6	153.7 (149.2-158.3)	21.1	4232	134.7	142.3 (137.9-146.6)	18.9
Aizawl, Mizoram (2015-2019)	78.6	21.4	2253	202.0	198.4 (190.1-206.7)	26.1	2124	181.9	172.5 (165.0-179.9)	22.1
21										
West Arunachal, Arunachal Pradesh (2015-2019)^c^	25.8	74.2	1392	59.0	87.9 (82.9-92.8)	13.0	1214	52.6	75.1 (70.6-79.6)	10.2
Papumpare, Arunachal Pradesh (2015-2019)	54.9	45.1	515	93.7	163.8 (147.2-180.4)	23.1	473	83.2	136.4 (122.3-150.4)	18.6
22: Pasighat, Arunachal Pradesh (2015-2019)^d^	25.4	74.6	382	105.3	112.9 (101.4-124.3)	15.8	369	103.0	112.7 (101.0-124.5)	13.9
23										
Meghalaya, Meghalaya (2015-2019)^e^	24.9	75.1	5414	99.4	150.8 (146.7-155.0)	19.9	3352	61.0	82.7 (79.9-85.6)	11.5
East Khasi Hills, Meghalaya (2015-2019)	44.4	55.6	3269	139.5	191.8 (185.0-198.6)	24.8	2042	84.6	100.7 (96.3-105.2)	14.1
24										
Manipur state (2015-2019)	29.2	70.8	4166	48.7	50.6 (49.1-52.2)	7.9	5017	59.3	58.5 (56.8-60.1)	8.4
Imphal West, Manipur (2015-2019)	62.3	37.7	1263	90.6	77.5 (73.2-81.9)	11.6	1681	115.0	94.1 (89.5-98.7)	12.9
25: Nagaland, Nagaland (2015-2019)^f^	49.3	50.7	1604	77.0	100.9 (95.8-106.1)	14.5	1187	60.2	76.2 (71.6-80.7)	9.8
26: Kolkata, West Bengal (2015-2017)	100	0	8930	130.3	83.3 (81.5-85.1)	11.8	7814	119.7	79.9 (78.1-81.7)	10.6
27: Wardha district, Maharashtra (2015-2019)	32.5	67.5	2714	78.9	56.9 (54.7-59.1)	7.6	2860	87.3	62.3 (59.9-64.6)	8.0
28: Barshi Rural, Maharashtra (2015-2019)	0	100	779	55.8	40.4 (37.5-43.4)	5.8	870	69.9	50.4 (46.9-53.9)	6.7
29: Mumbai, Maharashtra (2015-2018)	100	0	27 866	102.9	85.5 (84.5-86.5)	11.7	28 703	120.5	90.8 (89.7-91.8)	12.3
30: Aurangabad, Maharashtra (2015-2019)	100	0	2153	58.3	57.0 (54.5-59.4)	7.7	2239	64.2	61.0 (58.5-63.6)	8.3
31: Osmanabad and Beed, Maharashtra (2015-2019)	18.7	81.3	4570	37.4	30.4 (29.5-31.3)	4.0	5433	48.9	36.3 (35.3-37.3)	5.0
32: Pune, Maharashtra (2015-2019)	100	0	11 314	72.0	67.5 (66.2-68.7)	9.6	13 011	91.3	79.9 (78.5-81.3)	11.3
33: Nagpur, Maharashtra (2015-2019)	100	0	6235	89.4	72.0 (70.2-73.8)	9.6	6360	93.1	72.4 (70.6-74.3)	9.1
34: Sindhudurg, Maharashtra (2017-2018)	12.6	87.4	402	49.9	31.2 (27.9-34.5)	4.2	462	58.1	36.6 (33.0-40.3)	4.5
35: Ratnagiri, Maharashtra (2017-2018)	16.3	83.7	855	60.4	41.0 (38.1-43.9)	5.5	1067	68.7	45.7 (42.7-48.6)	5.6
36: Hyderabad district, Telangana (2015-2018)	100	0	7868	96.4	92.7 (90.6-94.8)	12.8	9966	126.4	123.8 (121.3-126.3)	16.7
37: Visakhapatnam, Andhra Pradesh (2017-2018)	52.5	47.5	2149	47.3	39.5 (37.8-41.2)	5.3	3137	68.2	53.4 (51.5-55.3)	6.9
38: Bangalore, Karnataka (2015-2018)	100	0	21 321	103.9	99.9 (98.5-101.2)	14.3	25 331	132.5	121.0 (119.5-122.6)	16.5
39										
Malabar, Kerala (2015-2018)	56.6	43.4	12 941	172.8	119.9 (117.8-122.0)	17.3	11 277	131.9	87.1 (85.4-88.8)	11.5
Kannur, Kerala (2015-2018)	65.0	35.0	9387	195.8	127.4 (124.7-130.0)	18.3	8282	148.5	92.9 (90.8-95.0)	12.2
Kasaragod, Kerala (2015-2018)	38.9	61.1	3469	132.7	105.0 (101.5-108.6)	15.4	2913	101.4	75.7 (72.9-78.5)	10.0
40: Kollam district, Kerala (2015-2019)	45.0	55.0	11 919	191.4	114.8 (112.6-116.9)	16.6	11 784	165.6	101.2 (99.3-103.2)	13.0
41										
Thiruvananthapuram district, Kerala (2015-2019)	53.7	46.3	14 966	188.3	114.4 (112.5-116.3)	15.9	16 529	188.3	114.8 (112.9-116.7)	14.8
Pathanamthitta, Kerala (2019)^g^	11.0	89.0	1408	260.9	122.5 (115.3-129.6)	17.9	1316	209.4	108.1 (101.3-115.0)	14.3
Alappuzha, Kerala (2019)^g^	54.0	46.0	2360	233.2	125.5 (120.1-130.9)	18.7	2187	193.3	106.1 (101.1-111.0)	14.2
42: Tamil Nadu state (2015-2017)	48.4	51.6	88 665	75.9	57.8 (57.4-58.1)	8.0	109 558	93.6	68.6 (68.1-69.0)	9.0
43: Chennai, Tamil Nadu (2015-2018)^h^	100	0	12 630	131.2	99.6 (97.8-101.4)	13.3	14 728	151.7	111.0 (109.1-112.8)	14.9

Abbreviations: AAIR, age-adjusted rate per 100 000; CIR, crude incidence rate; ISP, Indian standard population; PBCR, population-based cancer registry; SN, serial number; UT, Union Territory.

^a^ Muzaffarpur covered Motipur, Kanti, Musahari, Sakra, Muraul, and Muzaffarpur Municipal Corporation.

^b^ Karimganj covered Karimganj, Hailakandi, and Dima Hasao.

^c^ West Arunachal covered Tawang, West Kameng, East Kameng, Upper Subansiri, Lower Subansiri, Kurung Kumey, Papumpare, and West Siang.

^d^ Pasighat covered East Siang and Upper Siang.

^e^ Meghalaya covered East Khasi Hills, West Khasi Hills, Jaintia Hills, and Ri Bhoi Districts.

^f^ Nagaland covered Kohima and Dimapur districts; Malabar covered Kasaragod, Mahe, and Kannur.

^g^ Pathanamthitta and Alappuzha were expanded districts of Thiruvananthapuram PBCR.

^h^ Chennai is part of the Tamil Nadu State and presented as a separate PBCR.

**Figure 1.**
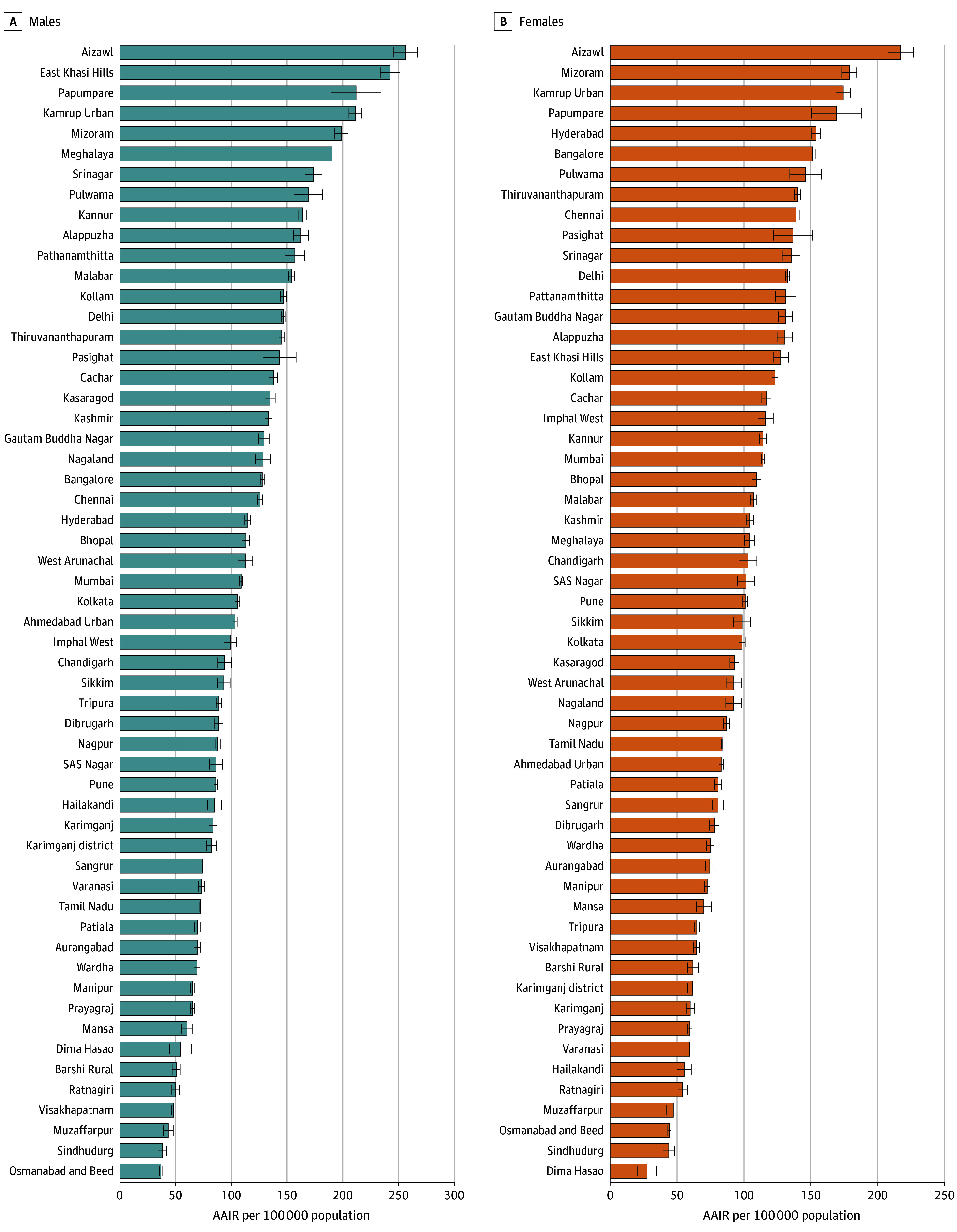
Comparison of All Cancer Sites’ Age-Adjusted Incidence Rates (AAIRs) of All Population-Based Cancer Registries (PBCRs), 2015 to 2019 Comparison of AAIR across the population was performed based on the World Standard Population (WSP; codes C00-C97 from the *International Statistical Classification of Diseases and Related Health Problems*, *10th Revision*). Error bars represent 95% CIs. A comparison of AAIR for selected leading sites of cancer across PBCRs is given in eFigure 1 in Supplement 1.

Figure 1 compares the AAIR per 100 000 population for all cancers (*ICD-10* codes C00-C97) across PBCRs. Aizawl reported the highest AAIR (using the WSP) in both males (256.1; 95% CI, 245.2-267.0) and females (217.2; 95% CI, 207.6-226.7), while the lowest rates were observed in Osmanabad and Beed for males (36.8; 95% CI, 35.7-37.9) and in Dima Hasao for females (27.6; 95% CI, 20.6-34.7). AAIRs varied regionally, with 6 northeastern populations having higher rates, followed by Srinagar (173.7; 95% CI, 166.0-181.3), Pulwama (168.9; 95% CI, 156.1-181.7), and Kannur (163.8; 95% CI, 160.4-167.1) among males, while among females Hyderabad ranked in fifth place with an AAIR of 153.8 (95% CI, 150.7-157.0), trailing the northeastern regions. Metropolitan cities such as Delhi (146.7; 95% CI, 145.1-148.3) and Chennai (125.7; 95% CI, 123.5-127.9), had higher AAIRs than Barshi Rural (50.6; 95% CI, 46.9-54.2) among males.

eFigure 1 in Supplement 1 depicts the comparison of AAIR for selected leading sites of cancer. Breast cancer had the highest AAIR in Hyderabad (54.0; 95% CI, 52.1-55.8) and the lowest in Dima Hasao (4.8; 95% CI, 1.8-7.7). Cervical cancer had the highest AAIR in Aizawl (27.1; 95% CI, 23.9-30.2), while the lowest AAIR was observed in Kashmir (1.6; 95% CI, 1.2-2.0). The highest AAIRs for lung cancer were observed in Srinagar (39.5; 95% CI, 35.8-43.2) for males and Aizawl (33.7; 95% CI, 29.7-37.7) for females. The highest AAIRs for oral cancer were observed in Ahmadabad Urban (33.6; 95% CI, 32.6-34.6) for males and East Khasi Hills (13.6; 95% CI, 11.6-15.5) for females. The highest AAIRs for prostate cancer were observed in Srinagar (12.7; 95% CI, 10.6-14.8) and Delhi (12.7; 95% CI, 12.2-13.2), followed by Gautam Buddha Nagar (11.7; 95% CI, 10.2-13.2).

Table 2 presents estimated cancer cases and rates (per 100 000 population) in India for 2024. The estimated cancer incidence for 2024 was 1 562 099 cases; estimated cancer mortality, 874 404 cases. The estimated number of new cancer cases among males in India was 780 822 with a CIR of 107.4. The estimated number of new cancer cases for females was 781 277 with a CIR of 113.3. The most common cancers in males consisted of mouth cancer (113 249 [CIR, 15.6]), followed by lung cancer (74 763 [CIR, 10.3]), and prostate cancer (49 998 [CIR, 6.9]). Among females, the most common cancers were breast (238 085 [CIR, 34.5]), cervix (78 499 [CIR, 11.4]), and ovarian (48 984 [CIR, 7.1]). Female genital system cancers were estimated to account for 171 497 cases (CIR, 24.9). In males, cancers of the oral cavity and pharynx were estimated to contribute to 217 327 cases (CIR, 29.9).

**Table 2.** Estimated Incidence of Cancer in India, 2024

Site	Males			Females			Both sexes		
	Cases, No.	CIR per 100 000 population	Cumulative risk of cancer, aged 0-74 y, %	Cases, No.	CIR per 100 000 population	Cumulative risk of cancer, aged 0-74 y, %	Cases, No.	CIR per 100 000 population	Cumulative risk of cancer, aged 0-74 y, %
All	780 822	107.4	11.6	781 277	113.3	11.1	1 562 099	110.3	11.3
Oral cavity and pharynx									
Overall	217 327	29.9	3.2	63 671	9.2	1.0	280 998	19.8	2.1
Tongue	61 859	8.5	0.9	21 351	3.1	0.3	83 210	5.9	0.6
Mouth	113 249	15.6	1.6	28 093	4.1	0.4	141 342	10.0	1.0
Pharynx	2272	0.3	<0.1	761	0.1	<0.1	3033	0.2	<0.1
Other oral cavity	39 947	5.5	0.7	13 466	2.0	0.2	53 413	3.8	0.4
Digestive system									
Overall	185 779	25.5	3.1	122 691	17.8	1.9	308 470	21.8	2.5
Esophagus	36 470	5.0	0.6	22 296	3.2	0.4	58 766	4.1	0.5
Stomach	32 401	4.5	0.5	17 833	2.6	0.3	50 234	3.5	0.4
Small intestine	3038	0.4	<0.1	2412	0.3	<0.1	5450	0.4	<0.1
Colon	24 906	3.4	0.4	18 325	2.7	0.3	43 231	3.1	0.3
Rectum	23 336	3.2	0.4	18 166	2.6	0.3	41 502	2.9	0.3
Anus, anal canal	2887	0.4	<0.1	1782	0.3	<0.1	4669	0.3	<0.1
Liver and intrahepatic bile duct	33 316	4.6	0.6	11 680	1.7	0.2	44 996	3.2	0.4
Gallbladder and other biliary	14 402	2.0	0.2	22 238	3.2	0.3	36 640	2.6	0.3
Pancreas	15 023	2.1	0.3	7959	1.2	0.1	22 982	1.6	0.2
Respiratory system									
Overall	106 094	14.6	1.9	36 447	5.3	0.6	142 541	10.1	1.2
Larynx	26 901	3.7	0.5	3242	0.5	<0.1	30 143	2.1	0.3
Lung and bronchus	74 763	10.3	1.3	30 446	4.4	0.5	105 209	7.4	0.9
Other respiratory organs	4430	0.6	0.1	2759	0.4	<0.1	7189	0.5	0.1
Bones and joints	7490	1.0	0.1	5391	0.8	0.1	12 881	0.9	0.1
Soft tissue	7426	1.0	0.1	6220	0.9	0.1	13 646	1.0	0.1
Skin (excluding basal and squamous)									
Overall	13 308	1.8	0.2	10 579	1.5	0.2	23 887	1.7	0.2
Melanoma of the skin	3619	0.5	0.1	2857	0.4	<0.1	6476	0.5	0.1
Other nonepithelial skin	9689	1.3	0.2	7722	1.1	0.1	17 411	1.2	0.1
Breast	5820	0.8	0.1	238 085	34.5	3.4	243 905	17.2	1.8
Genital system									
Overall	61 632	8.5	1.0	171 497	24.9	2.6	NA	NA	NA
Uterine cervix	NA	NA	NA	78 499	11.4	1.2	NA	NA	NA
Uterine corpus	NA	NA	NA	34 876	5.1	0.6	NA	NA	NA
Ovary	NA	NA	NA	48 984	7.1	0.7	NA	NA	NA
Vulva	NA	NA	NA	2601	0.4	<0.1	NA	NA	NA
Vagina and other genital, female	NA	NA	NA	6266	0.9	0.1	NA	NA	NA
Placenta	NA	NA	NA	271	0.0	<0.1	NA	NA	NA
Prostate	49 998	6.9	0.9	NA	NA	NA	NA	NA	NA
Testis	5379	0.7	0.1	NA	NA	NA	NA	NA	NA
Penis and other genital, male	6255	0.9	0.1	NA	NA	NA	NA	NA	NA
Urinary system									
Overall	33 382	4.6	0.5	11 442	1.7	0.2	44 824	3.2	0.4
Urinary bladder	20 015	2.8	0.3	5743	0.8	0.1	25 758	1.8	0.2
Kidney and renal pelvis	13 058	1.8	0.2	5506	0.8	0.1	18 564	1.3	0.1
Ureter and other urinary organs	309	0.0	<0.1	193	0.0	<0.1	502	0.0	<0.1
Eye and orbit	1124	0.2	<0.1	922	0.1	<0.1	2046	0.1	<0.1
Brain and other nervous system	21 673	3.0	0.3	12 940	1.9	0.2	34 613	2.4	0.2
Endocrine system									
Overall	9679	1.3	0.1	26 939	3.9	0.3	36 618	2.6	0.2
Thyroid	9003	1.2	0.1	26 261	3.8	0.3	35 264	2.5	0.2
Adrenal gland	676	0.1	<0.1	678	0.1	<0.1	1354	0.1	<0.1
Lymphoma									
Overall	29 643	4.1	0.4	17 517	2.5	0.3	47 160	3.3	0.3
Hodgkin lymphoma	6143	0.8	0.1	3350	0.5	<0.1	9493	0.7	0.1
Non-Hodgkin lymphoma	23 215	3.2	0.4	14 040	2.0	0.2	37 255	2.6	0.3
Malignant immunoproliferative disease	285	0.0	<0.1	127	0.0	<0.1	412	0.0	<0.1
Multiple myeloma	10 769	1.5	0.2	7830	1.1	0.1	18 599	1.3	0.2
Leukemia									
Overall	31 065	4.3	0.4	21 461	3.1	0.3	52 526	3.7	0.3
Lymphoid leukemia	12 842	1.8	0.1	7387	1.1	0.1	20 229	1.4	0.1
Myeloid leukemia	15 358	2.1	0.2	12 131	1.8	0.2	27 489	1.9	0.2
Leukemia unspecified	2865	0.4	<0.1	1943	0.3	<0.1	4808	0.3	<0.1
Other and unspecified primary sites	38 611	5.3	0.6	27 645	4.0	0.4	66 256	4.7	0.5

Abbreviations: CIR, crude incidence rate; NA, not applicable.

Table 3 presents India’s estimated cancer mortality cases and rates (per 100 000 population) in 2024. The estimated number of cancer mortality cases among males in India was 460 191 with a CMR of 63.3. For females, the estimated number of cancer mortality cases was 414 213 with a CMR of 60.1. The highest estimates of deaths from cancer among males were digestive system cancers (128 695 [CMR, 17.7]), followed by oral cavity and pharynx cancers (116 744 [CMR, 16.1]), and respiratory system cancers (76 686 [CMR, 10.5]). Among females, breast cancer had the highest estimated mortality (102 377 [CMR, 14.9]).

**Table 3.** Estimated Mortality of Cancer in India, 2024

Site	Males		Females		Both sexes	
	Cases, No.	CMR per 100 000 population	Cases, No.	CMR per 100 000 population	Cases, No.	CMR per 100 000 population
All	460 191	63.3	414 213	60.1	874 404	61.7
Oral cavity and pharynx						
Overall	116 744	16.1	37 418	5.4	154 162	10.9
Tongue	34 024	4.7	11 320	1.6	45 344	3.2
Mouth	55 491	7.6	16 856	2.4	72 347	5.1
Pharynx	2272	0.3	748	0.1	3020	0.2
Other oral cavity	24 957	3.4	8494	1.2	33 451	2.4
Digestive system						
Overall	128 695	17.7	83 308	12.1	212 003	15.0
Esophagus	27 716	3.8	16 277	2.4	43 993	3.1
Stomach	23 004	3.2	12 838	1.9	35 842	2.5
Small intestine	1428	0.2	1256	0.2	2684	0.2
Colon	12 702	1.7	10 449	1.5	23 151	1.6
Rectum	12 365	1.7	10 358	1.5	22 723	1.6
Anus, anal canal	1381	0.2	820	0.1	2201	0.2
Liver and intrahepatic bile duct	27 986	3.8	9810	1.4	37 796	2.7
Gallbladder and other biliary	9793	1.3	14 897	2.2	24 690	1.7
Pancreas	12 320	1.7	6603	1.0	18 923	1.3
Respiratory system						
Overall	76 686	10.5	27 779	4.0	104 465	7.4
Larynx	17 755	2.4	2239	0.3	19 994	1.4
Lung and bronchus	56 818	7.8	24 055	3.5	80 873	5.7
Other respiratory organs	2113	0.3	1485	0.2	3598	0.3
Bones and joints	3296	0.5	2534	0.4	5830	0.4
Soft tissue	3269	0.4	2797	0.4	6066	0.4
Skin (excluding basal and squamous)						
Overall	4549	0.6	4068	0.6	8617	0.6
Melanoma of the skin	1448	0.2	1518	0.2	2966	0.2
Other nonepithelial skin	3101	0.4	2550	0.4	5651	0.4
Breast	2326	0.3	102 377	14.9	104 703	7.4
Genital system						
Overall	26 933	3.7	86 219	12.5	NA	NA
Uterine cervix	NA	NA	42 392	6.1	NA	NA
Uterine corpus	NA	NA	8724	1.3	NA	NA
Ovary	NA	NA	29 880	4.3	NA	NA
Vulva	NA	NA	1091	0.2	NA	NA
Vagina and other genital, female	NA	NA	3861	0.6	NA	NA
Placenta	NA	NA	271	0.04	NA	NA
Prostate	23 498	3.2	NA	NA	NA	NA
Testis	1349	0.2	NA	NA	NA	NA
Penis and other genital, male	2086	0.3	NA	NA	NA	NA
Urinary system						
Overall	12 731	1.8	4910	0.7	17 641	1.2
Urinary bladder	7605	1.0	2527	0.4	10 132	0.7
Kidney and renal pelvis	4928	0.7	2264	0.3	7192	0.5
Ureter and other urinary organs	198	0.03	119	0.02	317	0.0
Eye and orbit	439	0.1	324	0.05	763	0.1
Brain and other nervous system	12 572	1.7	8154	1.2	20 726	1.5
Endocrine system						
Overall	2874	0.4	5973	0.9	8847	0.6
Thyroid	2520	0.3	5519	0.8	8039	0.6
Adrenal gland	354	0.05	454	0.1	808	0.1
Lymphoma						
Overall	14 726	2.0	9349	1.4	24 075	1.7
Hodgkin lymphoma	2152	0.3	1373	0.2	3525	0.2
Non-Hodgkin lymphoma	12 426	1.7	7892	1.1	20 318	1.4
Malignant immunoproliferative disease	148	0.02	84	0.01	232	0.0
Multiple myeloma	8507	1.2	5636	0.8	14 143	1.0
Leukemia						
Overall	19 592	2.7	15 121	2.2	34 713	2.5
Lymphoid leukemia	7064	1.0	4951	0.7	12 015	0.8
Myeloid leukemia	10 292	1.4	8614	1.2	18 906	1.3
Leukemia unspecified	2236	0.3	1556	0.2	3792	0.3
Other and unspecified primary sites	26 252	3.6	18 246	2.6	44 498	3.1

Abbreviations: CMR, crude mortality rate; NA, not applicable.

**Figure 2.**
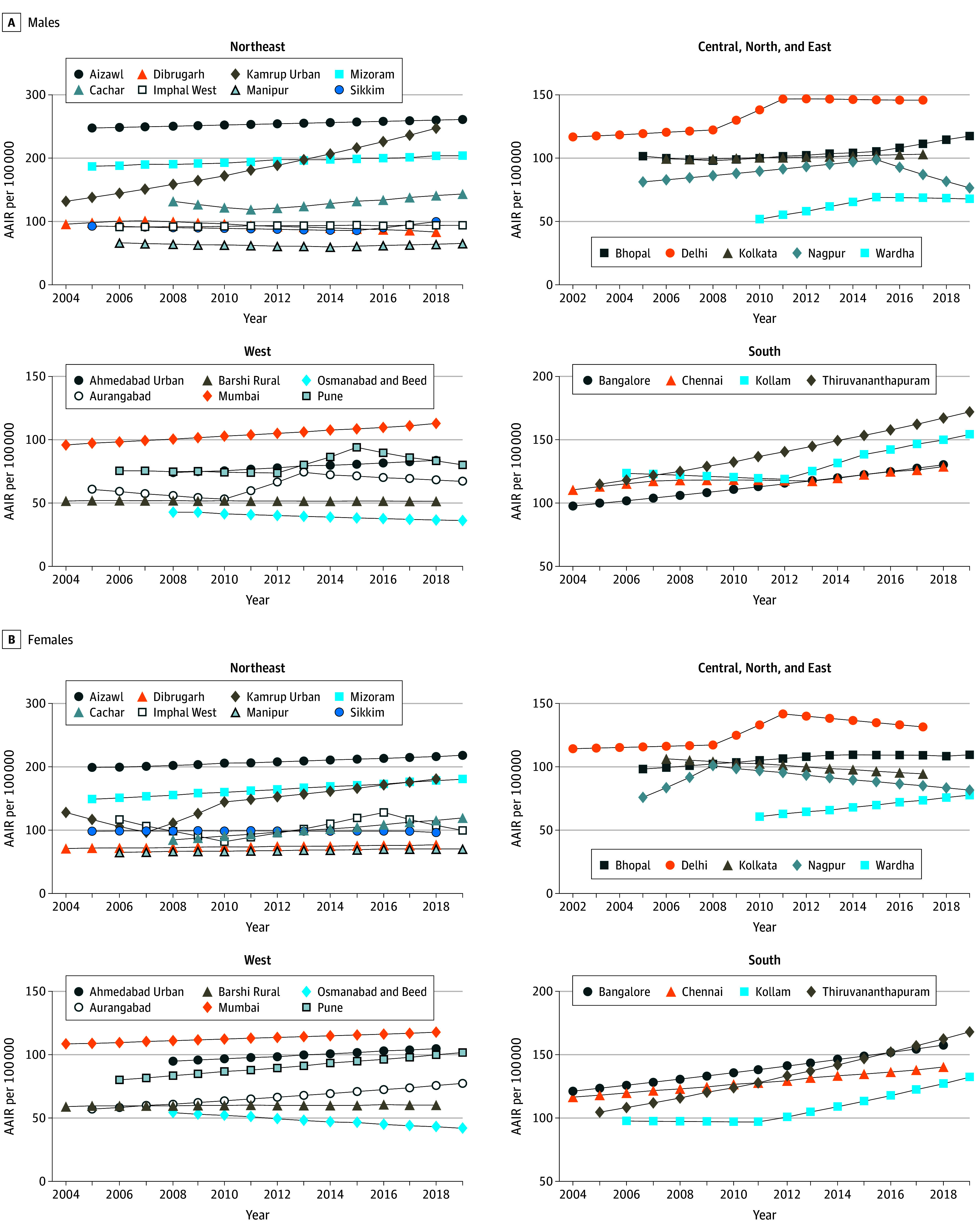
Trends in Age-Adjusted Incidence Rate (AAIR) for All Sites of Cancer, 2002 to 2019 Trends in AAIRs across the population were performed based on the World Standard Population (WSP), for all cancers combined; population-based cancer registries with more than 10 years of continuous data were included in the analysis, while those with fewer than 10 cases per year were excluded. Details regarding the average annual percent change values and trends in the AAIR for all cancer sites are provided in eTable 3 in Supplement 1. Additionally, trends in the AAIR from 2002 to 2019 for selected cancer sites are depicted in eFigure 2 in Supplement 1.

Figure 2 depicts the AAPC and trends in AAIR (2002-2019) for all sites of cancer, with PBCRs grouped by region. Among the 23 populations, a statistically significant increase in AAIR was observed in 9 populations among males and 14 among females. The highest increases in AAPC were observed in Kamrup Urban for males (3.3%; 95% CI, 2.3%-4.3%) and females (2.4%; 95% CI, −1.8% to 6.8%), Wardha for males (3.0%; 95% CI, 1.5%-4.6%) and females (2.7%; 95% CI, 1.0%-4.4%), and Thiruvananthapuram Taluk for males (2.9%; 95% CI, 2.4%-3.4%) and females (3.4%; 95% CI, 3.1%-3.8%) (eTable 3 in Supplement 1).

eFigure 2 in Supplement 1 depicts the trends in AAIR (2002-2019) and AAPC for the selected leading anatomical sites. Oral cancer showed significant increases in 14 PBCRs among males and 4 PBCRs among females. Meanwhile, the stomach cancer incidence rates decreased in both males and females, specifically in Aizawl (−3.8% [95% CI, −5.3% to −2.3%] and −5.9% [95% CI, −8.0% to −3.8%], respectively), Mizoram (−3.0% [95% CI, −4.3% to −1.8%] and −4.1% [95% CI, −5.4% to −2.9%], respectively), and Chennai (−2.0% [95% CI, −2.9% to −1.0%] and −1.4% [95% CI, −2.6% to −0.3%], respectively). The AAIR of lung cancer exhibited a statistically significant increase in 5 PBCRs for males and 9 PBCRs for females, including Kamrup Urban (3.8% [95% CI, 1.8%-5.8%] and 6.3% [95% CI, 2.9%-9.7%], respectively), Bangalore (3.7% [95% CI, 2.2%-5.2%] and 5.5% [95% CI, 3.2%-7.9%], respectively), Kollam (2.5% [95% CI, 1.5%-3.6%] and 3.9% [95% CI, 1.8%-6.1%], respectively), and Thiruvananthapuram Taluk (2.3% (95% CI, 1.0%-3.5%] and 6.1% [95% CI, 2.4%-10.0%], respectively). The AAIR of prostate cancer demonstrated statistically significant increases trends in 8 PBCRs.

eFigure 3 in Supplement 1 depicts the AAPC and trends in AAMR (2002-2019) for all sites of cancer across the selected PBCRs. The highest increases in AAPC were observed in Wardha at 8.5% (95% CI, 3.7%-13.6%) for males and 8.6% (95% CI, 5.0%-12.3%) for females and Mumbai at 2.6% (95% CI, 1.0%-4.2%) for females and 3.1% (95% CI, 1.7%-4.5%) for males (eTable 4 in Supplement 1).

eFigure 4 in Supplement 1 presents the top 3 leading cancer sites across the PBCRs. Among males, lung, mouth, stomach, esophagus, and prostate cancers were the most common cancers. For females, breast, cervical, and ovarian cancers were the most common.

eTable 5 in Supplement 1 presents mortality statistics across PBCRs, including number, CMR, AAMR (per 100 000 population), and M:I ratios. The Aizawl district, Mizoram, had the highest AAMR for males and females at 155.9 (95% CI, 147.3-164.5) and 96.4 (95% CI, 89.9-102.9), respectively. The M:I ratio for the leading cancer sites is presented in eTable 6 in Supplement 1, where variations by cancer site were observed across the PBCRs.

eTable 7 in Supplement 1 provides a comprehensive overview of the quality indicators for PBCRs across various geographical areas. Microscopic verification was more than 75% in all the PBCRs except in Mansa (72.4%), Dima Hasao (69.6%), and Varanasi (65.7%).

## Discussion

This cross-sectional study provides a comprehensive overview of cancer incidence and mortality in India for the varying period of 2015 to 2019 from 43 PBCRs. Our findings indicate that regions such as Aizawl, East Khasi Hills, Papumpare, Kamrup Urban, and Mizoram of northeastern India consistently recorded the highest incidence rates of cancer, in alignment with previous publications from the NCRP.^5,23^ Esophageal cancer was most prevalent in the northeastern region of the country. The analysis also indicated a higher incidence rate in urban areas compared with rural areas. The Delhi metropolitan area recorded an AAIR 3 times higher than that of Barshi Rural, a pattern consistent with findings from another study.^24^ A significant rise in cancer incidence was observed in most of the geographical areas studied. In India, the leading cancer sites were mouth for males and breast for females.

The analysis revealed a distinct pattern in the leading cancer sites across India. Among males, lung cancer emerged as the most frequently diagnosed cancer in the southern regions and metropolitan cities, including Visakhapatnam, Bangalore, Malabar, Kollam, Thiruvananthapuram, Chennai, and Delhi. A previous study^7^ found that patients in India tend to present with lung cancer about a decade earlier than those in western populations, with median age ranging from 54 to 70 years. Additionally, half of the patients were diagnosed with advanced-stage disease.^7^ A systematic review and meta-analysis on tobacco use^25^ revealed a significantly higher risk of respiratory system cancers (lung cancer), with an odds ratio of 4.97 (95% CI, 3.62-6.32). Mouth cancer is the predominant cancer site in the western (Ahmedabad Urban, Bhopal, Nagpur, and Wardha), central (Barshi Rural, Mumbai, Aurangabad, Osmanabad and Beed, Pune, Sindhudurg, and Ratnagiri), and certain northern (Prayagraj and Varanasi) regions. As tobacco and alcohol use are major risk factors, it is vital to promote widespread education about their harmful effects.^26,27^ Furthermore, quitline services and the implementation of early detection programs are critical for effective prevention and control.^28,29^

In India, breast, cervical, and ovarian cancers consistently ranked among the top 3 most common cancers in women, with disparities observed in survival rates for breast and cervical cancers.^30,31^ The increasing incidence of breast cancer and decreasing incidence of cervical cancer were more associated with generational shifts in risk factors than period effects.^32^ The significant variation in area-wise cancer incidence rates and cancer types in India highlights the need for tailored strategies to enhance cancer prevention, and control efforts. These insights may guide future studies on environmental and lifestyle risk factors in Indian populations. This heterogeneity underscores the importance of strengthening infrastructure and human resources both nationally and at the state level.^33^ Similar to India, Thailand and China have experienced significant increases in the estimated APC in all sites of cancer incidence during the past 15 years.^34^

A similar approach was used to estimate cancer cases for India by incorporating region-specific historical incidence trend data.^1,5,16^ In 2024, an estimated 1 562 099 new cancer cases were expected, with females having a higher estimated incidence rate than males. PBCRs with poor-quality indicators, as well as data from the year 2020 (due to the impact of COVID-19), were excluded from the estimation process.^35^ Worldwide, the COVID-19 pandemic has had a considerable effect on health care systems, including cancer registries. Our analysis of PBCRs with finalized 2020 data revealed a decline in cancer incidence rates (eTable 2 in Supplement 1). Investing in prevention strategies that address key cancer risk factors, including smoking, overweight and obesity, and infections, could prevent millions of cancer cases globally. These efforts will also generate substantial economic and societal benefits for countries.^36^

High-quality PBCRs in India, known for their comprehensive regional representation of geographic and demographic diversity, have been consistently included in the Cancer Incidence in Five Continents (CI5) volumes by the World Health Organization–International Agency for Research on Cancer, enhancing the generalizability of their findings to broader regional and national contexts.^37^ The latest CI5 volume XII featured data from 24 PBCRs in India, 19 of which were contributed by the Indian Council Medical Research–National Centre for Disease Informatics and Research, 3 from TMC, and 2 from the Tamil Nadu Cancer Registry Programme.^37^ Although mortality figures from various registries may be incomplete, the mortality data provided by Mumbai were relatively more consistent and complete over time. Therefore, estimated mortality cases for India were calculated by applying Mumbai’s M:I ratio to the estimated incidence of cancer cases, similar to the GCO method.^1^

Effective cancer control in India requires coordinated efforts, focusing on public awareness, prevention, and early detection. Awareness campaigns help reduce stigma and encourage timely health-seeking behavior. Beyond prevention, upgrading existing cancer care facilities and expanding services in high-incidence regions is vital to ensure equitable access to quality and affordable care. However, cancer care delivery faces challenges, including regional disparities, socioeconomic inequalities, low awareness, and varied health-seeking patterns. Addressing these issues requires a collaborative, data-driven approach to build equitable and accessible cancer care across India.^38^ The Indian government has strengthened cancer control through nationwide screening under the National Programme for Prevention and Control of Non-Communicable Diseases, financial protection through Ayushman Bharat Pradhan Mantri Jan Arogya Yojana for low-income groups, expansion of tertiary care with 19 state cancer institutes and 20 tertiary care centers, and plans to establish 200 district day care cancer centers by 2025 to 2026 to improve access to treatment.^39^

### Limitations

This study has limitations. Unlike incidence, mortality data are not uniformly captured across India, and underreporting of deaths across PBCRs may have occurred due to incorrect or incomplete certification of causes of death and limited coverage. Although PBCRs cover approximately 18% of India’s total population, registries cover a wide range of urban and rural areas across different states, providing a reasonably representative snapshot of the country’s cancer burden. Nonetheless, care must be taken when interpreting these results for regions not currently represented, as local epidemiological, environmental, and health care factors may influence the distribution and outcomes of cancer.

The study also revealed differing rates of quality indicators across the PBCRs, influenced by the level of cooperation and reporting from sources of registration. Additionally, 26 areas with mostly newer PBCRs reported a death certificate–only verification rate of less than 2%, and 3 areas—Nagpur, Muzaffarpur, and Hailakandi—exhibited other and unspecified site rates exceeding 10%. These challenges can be overcome by engaging the state governments to bridge the gap between the source of registration and cancer registries for comprehensive data collection.

## Conclusions

This cancer registry–based cross-sectional study from 43 PBCRs highlights the regional disparities in cancer incidence and mortality across India and the growing cancer burden. In India, oral, lung, and prostate cancers were the most prevalent among males, while breast, cervical, and ovarian cancers were common among females. This underscores a need to strengthen the ongoing efforts for cancer prevention and control measures to reduce the burden of cancer in India.

## Notes

Supplement 1. **eTable 1.** *ICD-10* Codes for Various Cancer Sites **eTable 2.** Comparison of Incidence Rate Between 2015-2019 and 2015-2020 **eFigure 1.** Comparison of Age-Adjusted Incidence Rates (AAIRs) for Selected Leading Sites of Cancer Across PBCRs (2015-2019) **eTable 3.** AAPC and Trends in Age-Adjusted Incidence Rate (2002-2019) for All Sites of Cancer **eFigure 2.** AAPC and Trends in Age-Adjusted Incidence Rate (2002-2019) for Selected Sites of Cancer **eFigure 3.** Trends in Age-Adjusted Mortality Rate (2002-2019) for All Sites of Cancer **eTable 4.** AAPC and Trends in Age-Adjusted Mortality Rate (2002-2019) for All Sites of Cancer **eFigure 4.** Top 3 Leading Sites of Cancers Based on the Relative Proportion in PBCRs in India (2015-2019) **eTable 5.** Cancer Mortality Cases: Number, Mortality-Incidence Ratio (M/I), and Rates (CMR and AAMR) per 100 000 by Sex in 43 PBCRs (2015-2019), India **eTable 6.** Mortality-to-Incidence Ratio of the Top Leading Cancer Sites Across 43 PBCRs (2015-2019) **eTable 7.** Data Quality Indicators: Number and Relative Proportion, All Sites of Cancer (2015-2019), Both Sexes

Supplement 2.
Data Sharing Statement
