# Supplementary material for: Cancer Incidence and Mortality Across 43 Cancer Registries in India
Source: JAMA Netw Open. 2025 Aug 20;8(8):e2527805. doi: 10.1001/jamanetworkopen.2025.27805 (PMC12368690; doi:10.1001/jamanetworkopen.2025.27805)
Supplement: Supplement 1. — eTable 1. ICD-10 Codes for Various Cancer Sites eTable 2. Comparison of Incidence Rate Between 2015-2019 and 2015-2020 eFigure 1. Comparison of Age-Adjusted Incidence Rates (AAIRs) for Selected Leading Sites of Cancer Across PBCRs (2015-2019) eTable 3. AAPC and Trends in Age-Adjusted Incidence Rate (2002-2019) for All Sites of Cancer eFigure 2. AAPC and Trends in Age-Adjusted Incidence Rate (2002-2019) for Selected Sites of Cancer eFigure 3. Trends in Age-Adjusted Mortality Rate (2002-2019) for All Sites of Cancer eTable 4. AAPC and Trends in Age-Adjusted Mortality Rate (2002-2019) for All Sites of Cancer eFigure 4. Top 3 Leading Sites of Cancers Based on the Relative Proportion in PBCRs in India (2015-2019) eTable 5. Cancer Mortality Cases: Number, Mortality-Incidence Ratio (M/I), and Rates (CMR and AAMR) per 100 000 by Sex in 43 PBCRs (2015-2019), India eTable 6. Mortality-to-Incidence Ratio of the Top Leading Cancer Sites Across 43 PBCRs (2015-2019) eTable 7. Data Quality Indicators: Number and Relative Proportion, All Sites of Cancer (2015-2019), Both Sexes [file jamanetwopen-e2527805-s001.pdf]

Supplementary Online Content

National Cancer Registry Programme Investigator Group. Patterns and trends in cancer incidence and mortality across 43 cancer registries in India. *JAMA Netw Open.* 2025;8(8):e2527805. doi:10.1001/jamanetworkopen.2025.27805

eTable 1. *ICD-10* Codes for Various Cancer Sites

eTable 2. Comparison of Incidence Rate Between 2015-2019 and 2015-2020

eFigure 1. Comparison of Age-Adjusted Incidence Rates (AAIRs) for selected leading sites of cancer Across PBCRs, 2015-2019

eTable 3. AAPC and Trends in Age-Adjusted Incidence Rate (2002-2019) for All Sites of Cancer

eFigure 2. AAPC and Trends in Age-Adjusted Incidence Rate (2002-2019) for Selected Sites of Cancer

eFigure 3. Trends in Age-Adjusted Mortality Rate (2002-2019) for All Sites of Cancer

eTable 4. AAPC and Trends in Age-Adjusted Mortality Rate (2002-2019) for All Sites of Cancer

eFigure 4. Top 3 Leading Sites of Cancers Based on the Relative Proportion in PBCRs in India (2015-2019)

eTable 5. Cancer Mortality Cases: Number, Mortality-Incidence Ratio (M/I), and Rates (CMR and AAMR) per 100 000 by Sex in 43 PBCRs (2015-2019), India

eTable 6. Mortality-to-Incidence Ratio of the Top Leading Cancer Sites Across 43 PBCRs (2015-2019)

eTable 7. Data Quality Indicators: Number and Relative Proportion, All Sites of Cancer (2015-2019), Both Sexes

This supplementary material has been provided by the authors to give readers additional information about their work.

© 2025 National Cancer Registry Programme Investigator Group. *JAMA Network Open*.

eTable 1. *ICD-10* Codes for Various Cancer Sites

ICD-10	Site
**C00-C97**	**All Sites**
**C00-C14**	**Oral cavity & pharynx**
C01-C02	Tongue
C03-C06	Mouth
C01-C06	Oral Cancer
C14	Pharynx
C00, C07-C08, C09-C13	Other oral cavity
**C15-C25**	**Digestive system**
C15	Oesophagus
C16	Stomach
C17	Small intestine
C18	Colon
C19-C20	Rectum
C21	Anus, anal canal
C22	Liver & intrahepatic bile duct
C23-C24	Gallbladder & other biliary
C25	Pancreas
**C30-38 + C45**	**Respiratory system**
C32	Larynx
C33-C34	Lung & bronchus
C30-C31, C37-C38, C45	Other respiratory organs
**C40-C41**	**Bones and joints**
**C46-C47+C49**	**Soft tissue**
**C43-C44**	**Skin (excluding basal & squamous)**
C43	Melanoma of the skin
C44	Other nonepithelial skin
**C50**	**Breast**

© 2025 National Cancer Registry Programme Investigator Group. *JAMA Network Open*.

eTable 1: ICD-10 codes for various cancer sites (Continued).

ICD-10	Site
**C51-C58, C60-C63**	**Genital System**
C53	Uterine cervix
C54	Uterine corpus
C56	Ovary
C51	Vulva
C52, C57, C55	Vagina & other genital, female
C58	Placenta
C61	Prostate
C62	Testis
C60, C63	Penis & other genital, male
**C64-C68**	**Urinary system**
C67	Urinary bladder
C64-C65	Kidney & renal pelvis
C66, C68	Ureter & other urinary organs
**C69**	**Eye & orbit**
**C70-C72**	**Brain & other nervous system**
**C73-74**	**Endocrine system**
C73	Thyroid
C74	Adrenal Gland
**C81-88, C96**	**Lymphoma**
C81	Hodgkin lymphoma
C82-C85, C96	Non-Hodgkin lymphoma
C88	Malig Imn.Prol D
**C90**	**Multiple Myeloma**
**C91-95**	**Leukemia**
C91	Lymphoid Leuk.
C92-C94	Myeloid Leukaemia
C95	Leukaemia Uns
**C26, C39, C48, C75, C76, C77, C78, C79, C80, C97**	**Other & unspecified primary sites**

Abbreviations: ICD-10, International Classification of Diseases, Tenth Revision; Malig Imn Prol D: Malignant immunoproliferative disease.

© 2025 National Cancer Registry Programme Investigator Group. *JAMA Network Open*.

eTable 2. Comparison of Incidence Rate Between 2015-2019 and 2015-2020

PBCR	2015-2019			2015-2020			
	Average No.	CIR	AAIR (WSP)	Average No.	CIR	AAIR (WSP)	% change
**Males**							
Aurangabad	431	58.3	69.5	430	57.5	68.2	-1.4
Pune	2263	72.0	86.1	2196	68.8	81.7	-4.4
Nagpur[a]	1247	89.4	87.8	1184	84.3	82.3	-5.7
Barshi rural	156	55.8	50.6	159	56.7	51.6	1.6
Meghalaya	1083	99.4	190.2	1079	97.9	187.4	-1.5
East Khasi hills	654	139.5	242.3	645	136.2	236.8	-2.4
Wardha	543	78.9	69.3	534	77.4	67.7	-1.9
West Arunachal	278	59.0	112.5	277	57.7	109.7	-2.2
Papumpare	103	93.7	211.9	103	92.1	208.6	-1.7
Bhopal (16-20)	928	98.2	113	966	97.0	110.9	-1.2
**Females**							
Aurangabad	448	64.2	74.5	443	62.5	72.0	-2.6
Pune[a]	2602	91.3	100.8	2502	86.3	95.1	-5.5
Nagpur[a]	1272	93.2	86.8	1202	87.3	81.0	-6.3
Barshi rural	174	69.9	61.8	173	69.0	60.8	-1.3
Meghalaya	671	61.0	104.1	679	60.9	103.8	-0.2
East Khasi hills	409	84.6	127.5	418	85.6	128.9	1.2
Wardha	572	87.3	74.8	553	84.2	71.8	-3.6
West Arunachal	243	52.6	92.5	246	52.3	92.8	-0.6
Papumpare	95	83.2	169.1	96	82.8	174.4	-0.5
Bhopal (16-19)	861	97.3	109.4	900	96.3	107.7	-1.0

Significance between Crude rates of 2015-2019, and 2015-2020 using Ratio test.

[a] *Statistically significant ($p<0.05$)*

Abbreviation: AAIR, age-adjusted incidence rate; CIR, crude incidence rate; WSP- world standard population

Note: PBCRs with complete 2020 data were taken for comparison.

© 2025 National Cancer Registry Programme Investigator Group. *JAMA Network Open*.

eFigure 1. Comparison of Age-Adjusted Incidence Rates (AAIRs) for selected leading sites of cancer Across PBCRs, 2015-2019

—A.Breast Cancer (ICD-10:C50) - Females

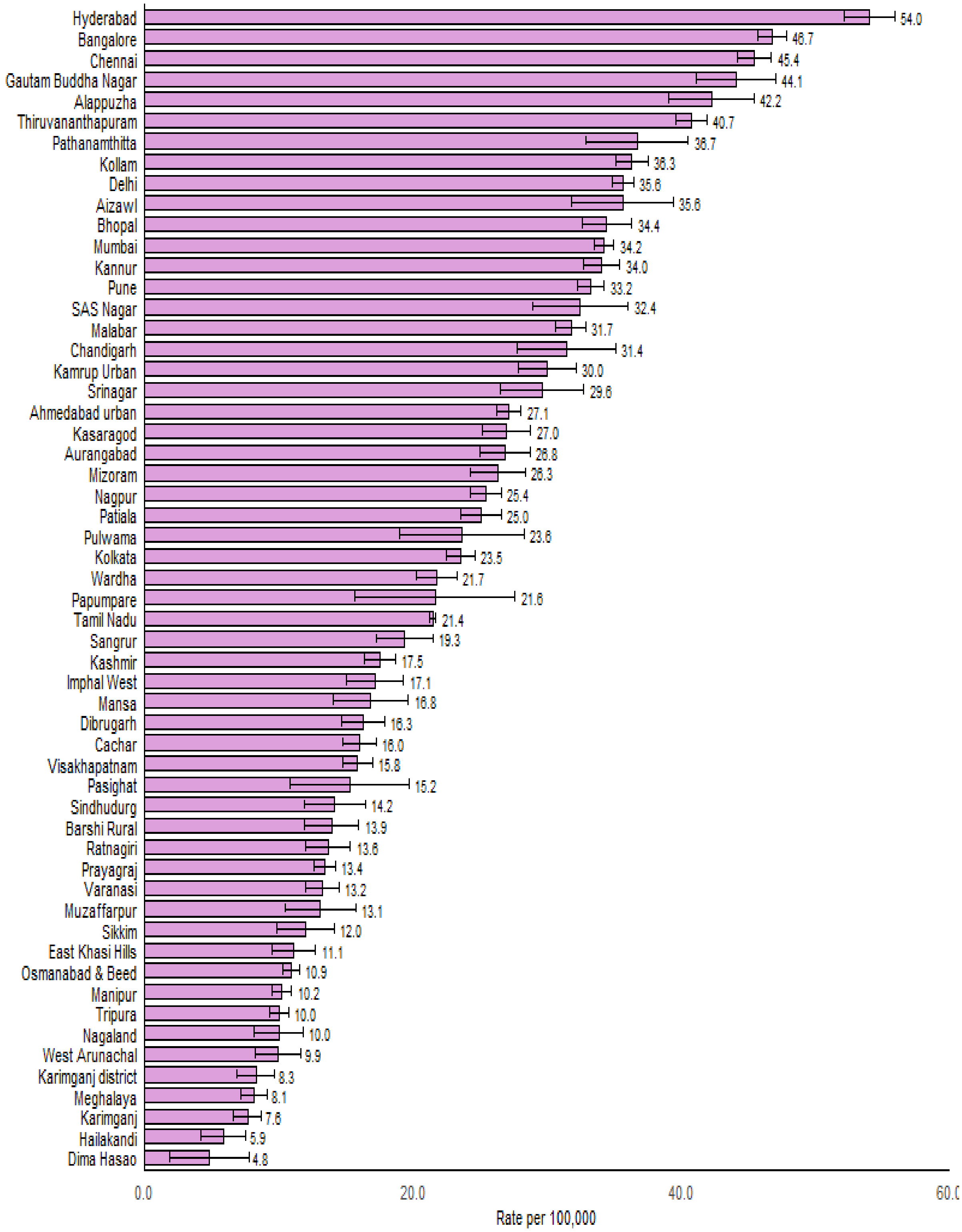

© 2025 National Cancer Registry Programme Investigator Group. *JAMA Network Open*.

B. Cervix Cancer (ICD 10: C53)

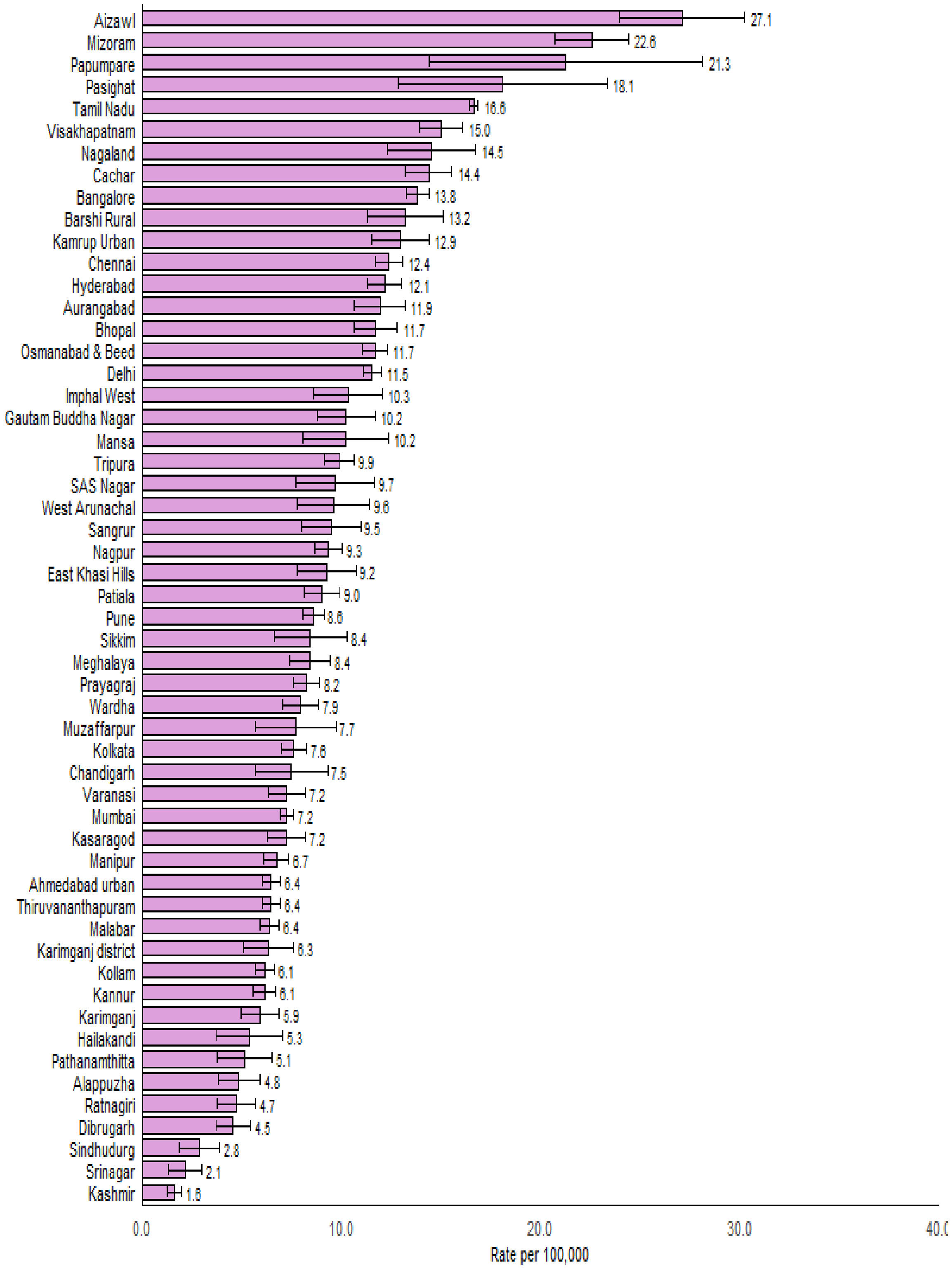

© 2025 National Cancer Registry Programme Investigator Group. *JAMA Network Open*.

–C.Lung Cancer (ICD 10: C33-C34)- Females

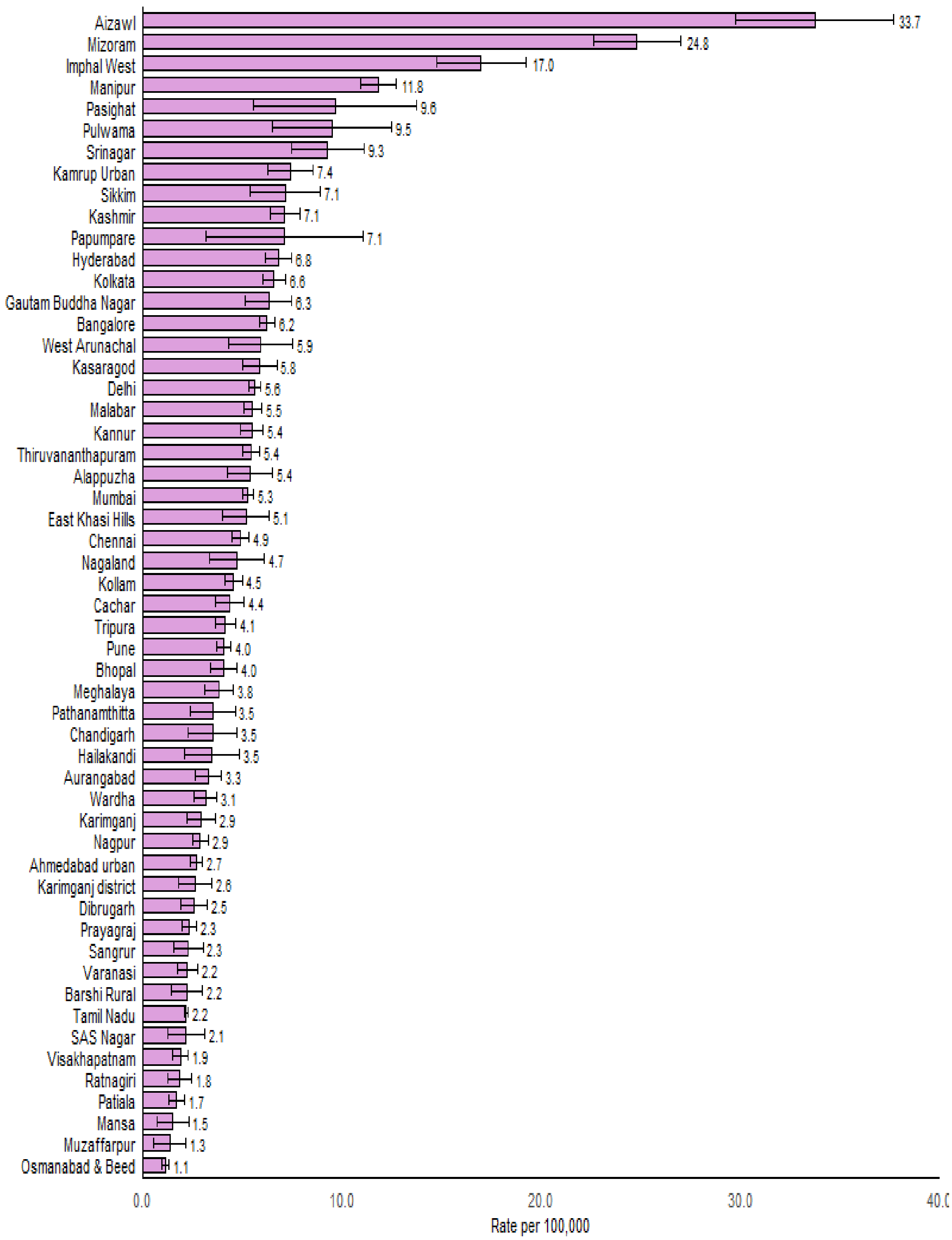

© 2025 National Cancer Registry Programme Investigator Group. *JAMA Network Open*.

D. Lung Cancer (ICD 10: C33-C34) - Males

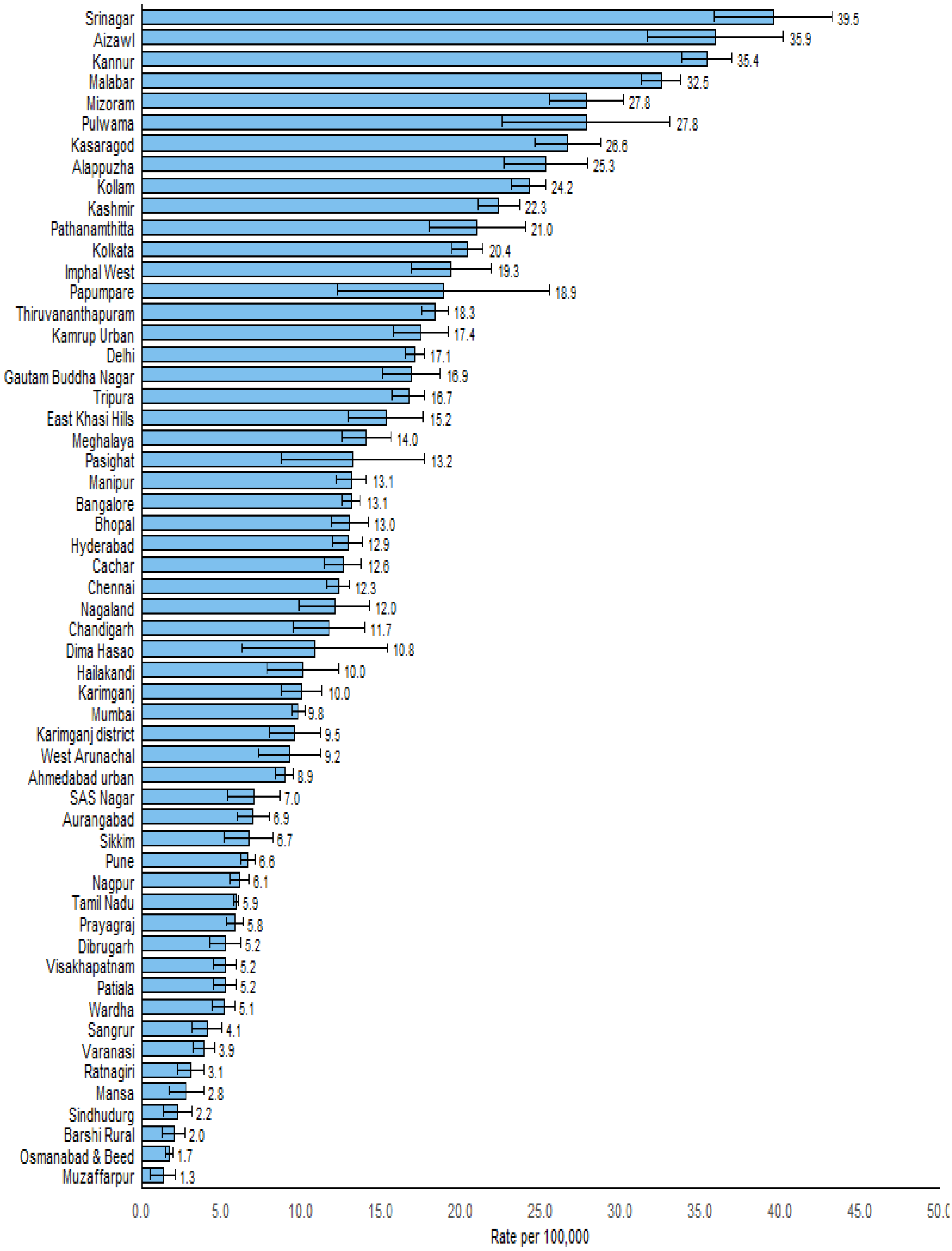

© 2025 National Cancer Registry Programme Investigator Group. *JAMA Network Open*.

E. Oral Cancer (ICD-10: C01-C06) - Females

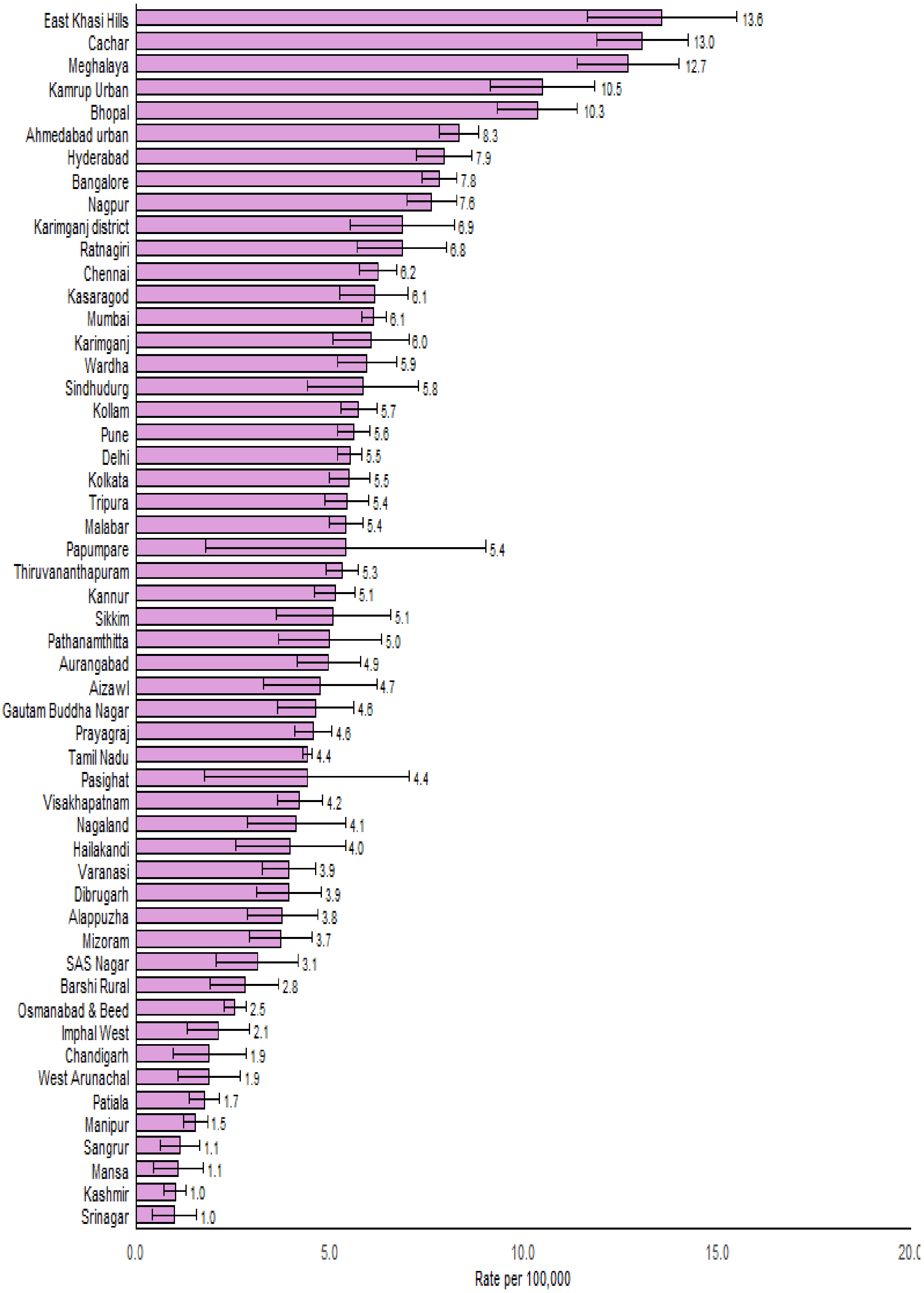

© 2025 National Cancer Registry Programme Investigator Group. *JAMA Network Open*.

F. Oral Cancer (ICD-10: C01-C06) - Males

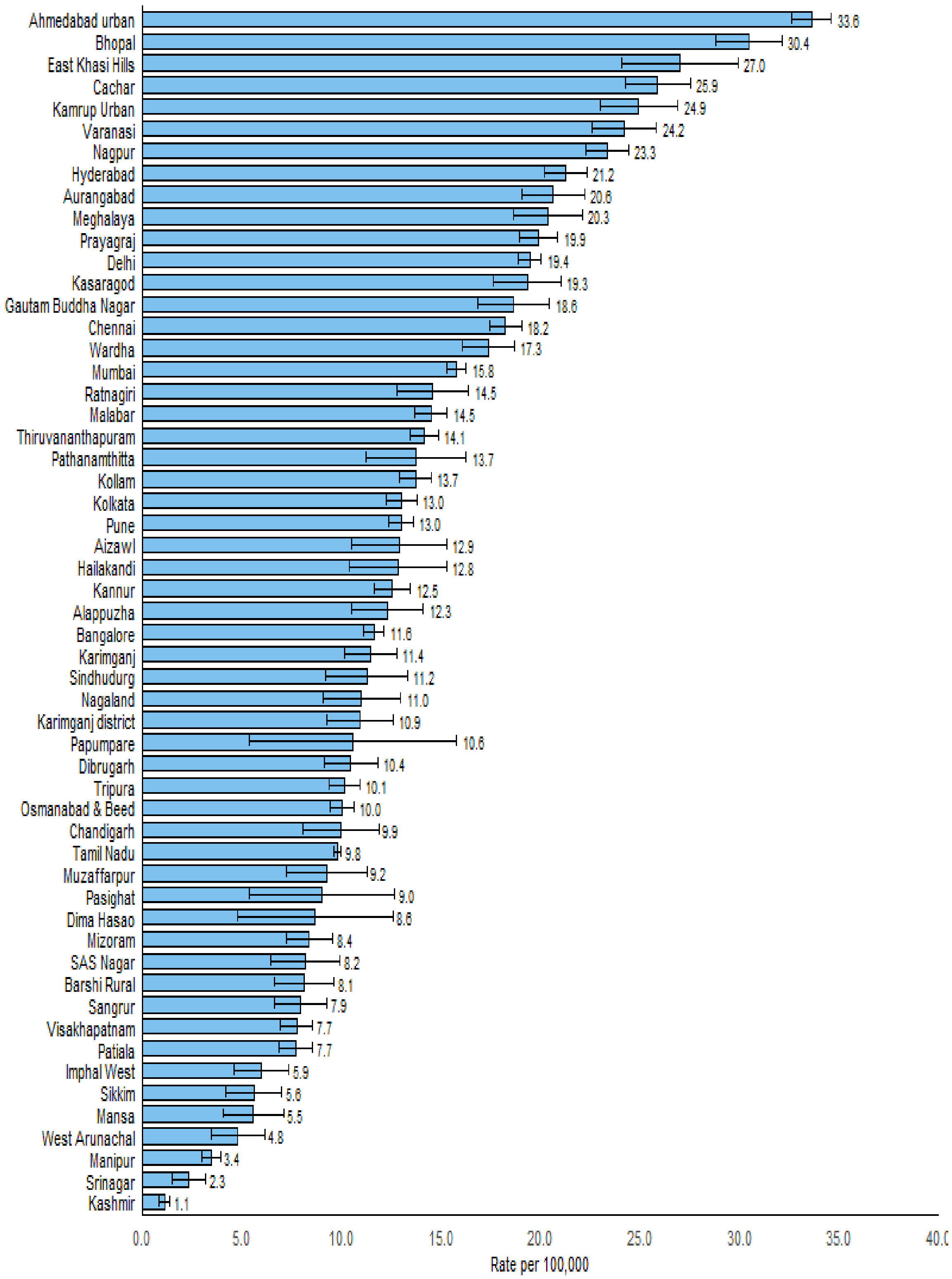

© 2025 National Cancer Registry Programme Investigator Group. *JAMA Network Open*.

–G.Stomach Cancer (ICD 10: C16)- Females

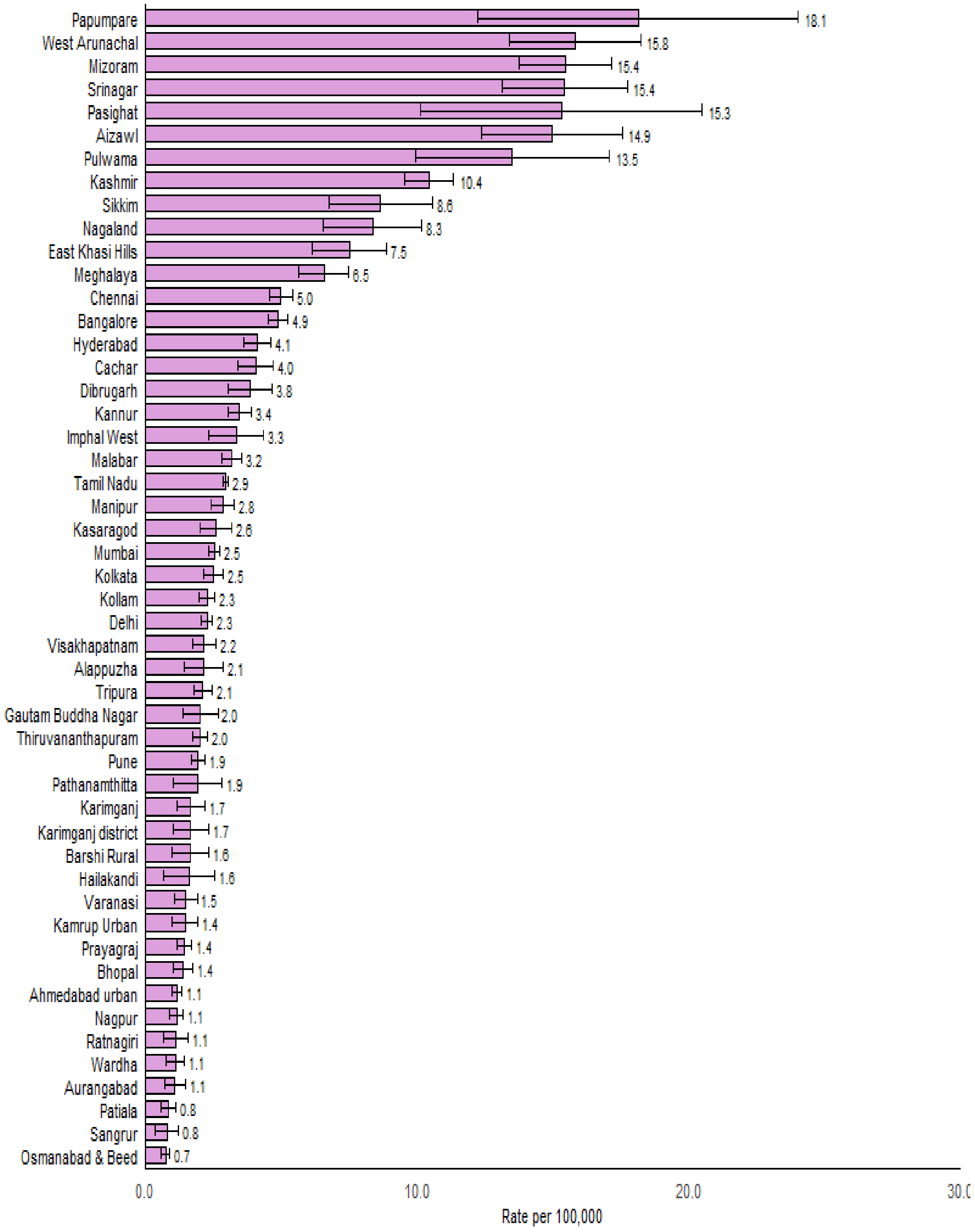

© 2025 National Cancer Registry Programme Investigator Group. *JAMA Network Open*.

H. Stomach Cancer (ICD-10:C16)- Males

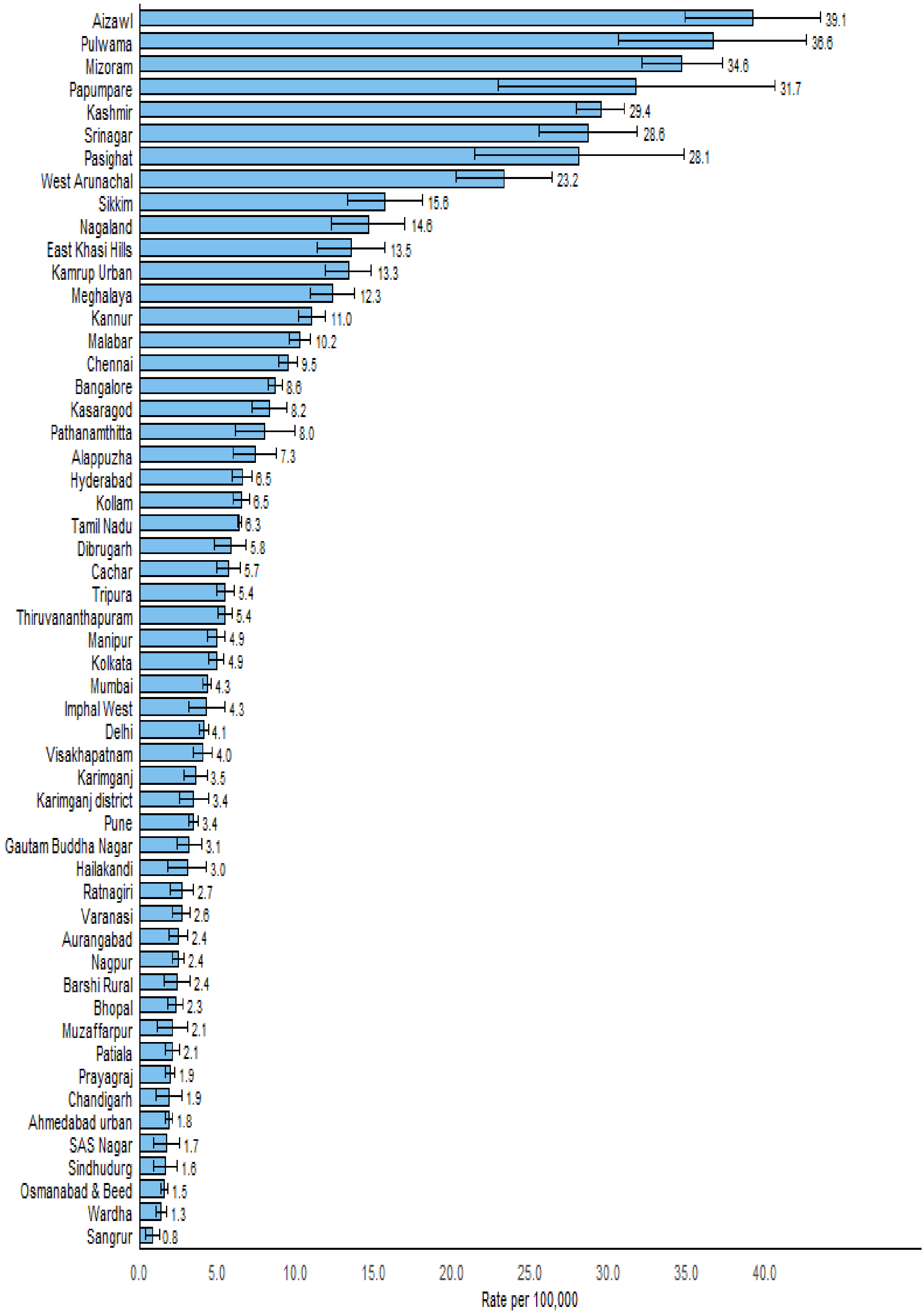

© 2025 National Cancer Registry Programme Investigator Group. *JAMA Network Open*.

I. Prostate Cancer (ICD-10:C61)

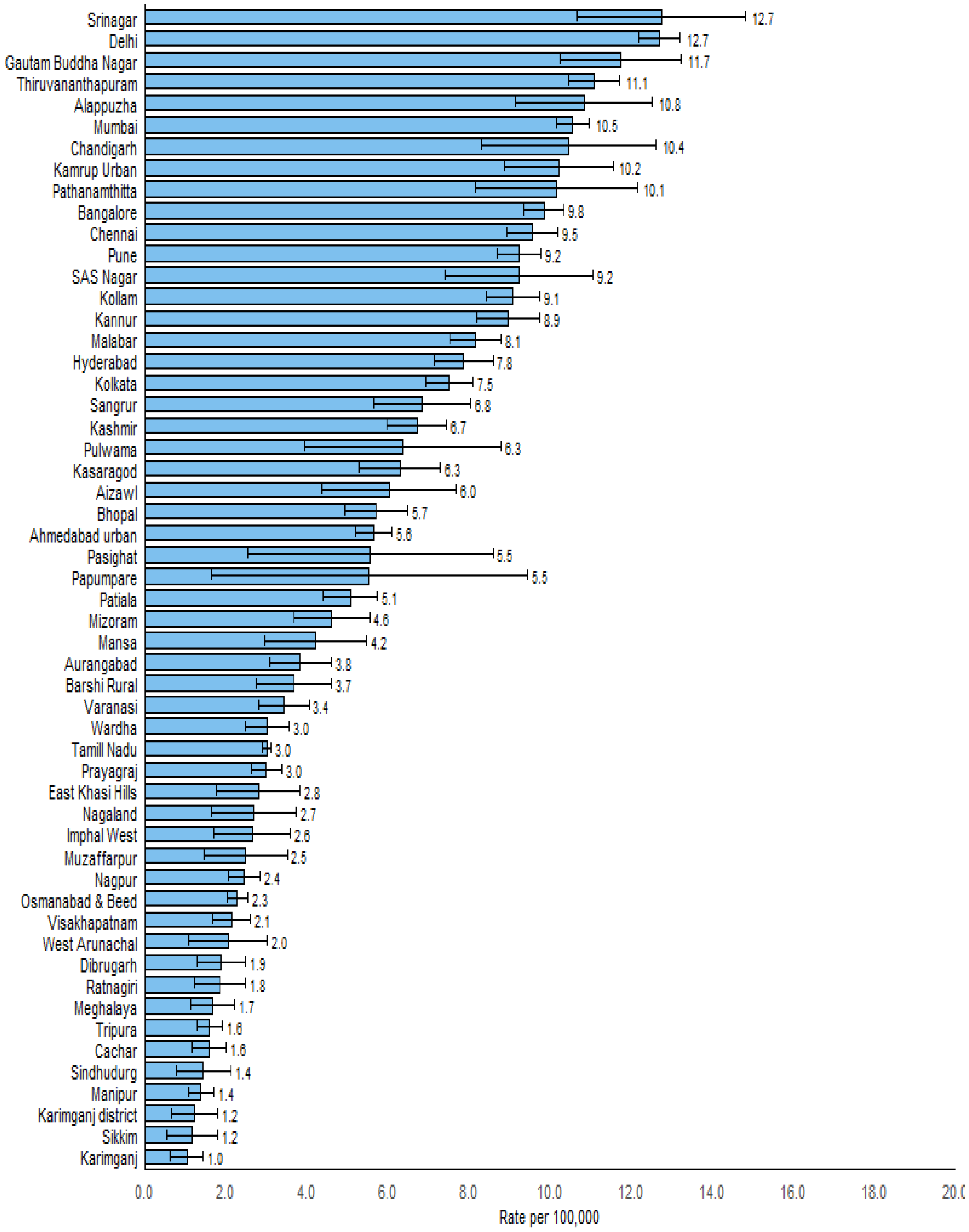

Note: Comparison of Age-adjusted incidence rate (AAIR) across the population was performed based on the World Standard Population (WSP). Error bars represent 95% confidence intervals.

© 2025 National Cancer Registry Programme Investigator Group. *JAMA Network Open*.

eTable 3. AAPC and Trends in Age-Adjusted Incidence Rate (2002-2019) for All Sites of Cancer

Males

Registry	All years		Trend 1			Trend 2			Trend 3		
	AAPC, % (95% CI)	p-value	Years	APC, % (95% CI)	p-value	Years	APC, % (95% CI)	p-value	Years	APC, % (95% CI)	p-value
**Aizwal**	0.4 (-0.5 to 1.3)	0.39	2005 - 2019	0.4 (-0.5 to 1.3)	0.39	NA			NA		
**Cachar**	0.8 (-1.5 to 3.2)	0.48	2008 - 2011	-3.4 (-11.6 to 5.6)	0.39	2011 - 2019	2.5 (0.5 to 4.5)	**0.02**	NA		
**Dibrugarh**	-0.8 (-1.6 to -0.1)	**0.03**	2004 - 2007	1.8 (-1.9 to 5.6)	0.31s	2007 - 2018	-1.6 (-2.0 to -1.1)	**<0.001**	NA		
**Imphal west**	0.2 (-1.3 to 1.8)	0.73	2006 - 2019	0.2 (-1.3 to 1.8)	0.73	NA			NA		
**Kamrup urban**	3.3 (2.3 to 4.3)	**<0.001**	2004 - 2018	3.3 (2.3 to 4.3)	**<0.001**	NA			NA		
**Manipur**	-0.1 (-1.3 to 1.2)	0.88	2006 - 2014	-1.3 (-2.7 to 0.2)	0.08	2014 - 2019	1.8 (-1.2 to 4.8)	0.21	NA		
**Mizoram**	0.6 (-0.3 to 1.5)	0.17	2005 - 2019	0.6 (-0.3 to 1.5)	0.17	NA			NA		
**Sikkim**	0.5 (-0.9 to 1.9)	0.45	2005 - 2015	-0.8 (-1.8 to 0.1)	0.08	2015 - 2018	5.2 (-1.1 to 11.9)	0.10	NA		
**Bhopal**	1.1 (0.5 to 1.6)	**<0.001**	2005 - 2008	-1.1 (-3.1 to 0.9)	0.24	2008 - 2015	1.0 (0.3 to 1.7)	**0.01**	2015 - 2019	2.9 (1.6 to 4.2)	**0.001**
**Delhi**	1.5 (-0.4 to 3.5)	0.13	2002 - 2008	0.8 (-0.9 to 2.5)	0.33	2008 - 2011	6.3 (-4.0 to 17.7)	0.20	2011 - 2017	-0.1 (-1.8 to 1.6)	0.88
**Kolkata**	0.4 (-1.4 to 2.3)	0.61	2006 - 2017	0.4 (-1.4 to 2.3)	0.61	NA			NA		
**Nagpur**	-0.4 (-2.6 to 1.8)	0.70	2005 - 2015	2.0 (0.2 to 3.9)	**0.04**	2015 - 2019	-6.2 (-12.9 to 0.9)	0.08	NA		

© 2025 National Cancer Registry Programme Investigator Group. *JAMA Network Open*.

eTable 3. AAPC and Trends in Age-Adjusted Incidence Rate (2002-2019) for All Sites of Cancer (continued).

Males

Registry	All years	Trend 1			Trend 2				Trend 3		
	**AAPC, % (95% CI)**	**p-value**	**Years**	**APC, % (95% CI)**	**p-value**	**Years**	**APC, % (95% CI)**	**p-value**	**Years**	**APC, % (95% CI)**	**p-value**
**Wardha**	3.0 (1.5 to 4.6)	**<0.001**	2010 - 2015	5.9 (3.4 to 8.4)	**0.002**	2015 - 2019	-0.4 (-3.7 to 3.0)	0.78	NA		
**Ahmedabad Urban**	1.2 (0.1 to 2.4)	**0.04**	2008 - 2018	1.2 (0.1 to 2.4)	**0.04**	NA			NA		
**Aurangabad**	0.7 (-2.5 to 3.9)	0.68	2005 - 2010	-2.8 (-6.2 to 0.7)	0.10	2010 - 2013	11.5 (-4.9 to 30.8)	0.15	2013 - 2019	-1.5 (-4.1 to 1.2)	0.22
**Barshi rural**	-0.1 (-1.4 to 1.3)	0.94	2004 - 2018	-0.1 (-1.4 to 1.3)	0.94	NA			NA		
**Mumbai**	1.1 (0.5 to 1.7)	**0.001**	2004 - 2018	1.1 (0.5 to 1.7)	**0.001**	NA			NA		
**Osmanabad & Beed**	-1.5 (-2.8 to -0.3)	**0.02**	2008 - 2019	-1.5 (-2.8 to -0.3)	**0.02**	NA			NA		
**Pune**	0.4 (-1.9 to 2.8)	0.72	2006 - 2012	-0.5 (-2.3 to 1.4)	0.56	2012 - 2015	8.2 (-3.1 to 20.9)	0.13	2015 - 2019	-3.8 (-7.1 to -0.3)	**0.04**
**Bangalore**	2.2 (1.7 to 2.8)	**<0.001**	2004 - 2018	2.2 (1.7 to 2.8)	**<0.001**	NA			NA		
**Chennai**	1.2 (0.6 to 1.8)	**<0.001**	2004 - 2007	2.3 (0.2 to 4.4)	**0.04**	2007 - 2013	0.1 (-0.8 to 1.1)	0.75	2013 - 2018	1.8 (0.9 to 2.7)	**0.003**
**Kollam**	1.7 (0.6 to 2.8)	**0.003**	2006 - 2012	-0.7 (-1.5 to 0.2)	0.12	2012 - 2015	5.2 (-0.2 to 10.9)	0.06	2015 - 2019	2.7 (1.0 to 4.5)	**0.01**
**Thiruvananthapuram Taluk**	2.9 (2.4 to 3.4)	**<0.001**	2005 - 2019	2.9 (2.4 to 3.4)	**<0.001**	NA			NA		

© 2025 National Cancer Registry Programme Investigator Group. *JAMA Network Open*.

Females

Registry	All years		Trend 1			Trend 2			Trend 3		
	AAPC, % (95% CI)	p-value	Years	APC, % (95% CI)	p-value	Years	APC, % (95% CI)	p-value	Years	APC, % (95% CI)	p-value
**Aizawl**	0.7 (-0.3 to 1.7)	0.18	2005 - 2019	0.7 (-0.3 to 1.7)	0.18	NA			NA		
**Cachar**	3.1 (1.5 to 4.7)	**0.001**	2008 - 2019	3.1 (1.5 to 4.7)	**0.001**	NA			NA		
**Dibrugarh**	0.5 (-0.4 to 1.5)	0.22	2004 - 2018	0.5 (-0.4 to 1.5)	0.22	NA			NA		
**Imphal west**	-1.2 (-4.4 to 2.0)	0.46	2006 - 2010	-8.4 (-14.6 to -1.7)	**0.02**	2010 - 2016	7.6 (2.4 to 13.1)	**0.01**	2016 - 2019	-7.9 (-17.6 to 2.9)	0.11
**Kamrup urban**	2.4 (-1.8 to 6.8)	0.27	2004 - 2017	-9.2 (-18.1 to 0.5)	**0.06**	2007 - 2010	14.3 (-6.8 to 40.3)	**0.17**	2010 - 2018	2.8 (0.5 to 5.1)	**0.02**
**Manipur**	0.7 (0.2 to 1.2)	**0.01**	2006 - 2019	0.7 (0.2 to 1.2)	**0.01**	NA			NA		
**Mizoram**	1.4 (0.7 to 2.0)	**0.001**	2005 - 2019	1.4 (0.7 to 2.0)	**0.001**	NA			NA		
**Sikkim**	0.0 (-1.0 to 1.0)	0.96	2005 - 2018	0.0 (-1.0 to 1.0)	0.96	NA			NA		
**Bhopal**	0.8 (0.5 to 1.1)	**< 0.001**	2005 - 2019	0.8 (0.5 to 1.1)	**< 0.001**	NA			NA		
**Delhi**	1.5 (0.9 to 2.1)	**< 0.001**	2002 - 2017	1.5 (0.9 to 2.1)	**< 0.001**	NA			NA		
**Kolkata**	-1.0 (-2.7 to 0.7)	0.21	2006 - 2017	-1.0 (-2.7 to 0.7)	0.21	NA			NA		
**Nagpur**	0.5 (-2.1 to 3.3)	0.69	2005 - 2008	9.8 (-3.4 to 24.9)	0.13	2008 - 2019	-1.8* (-3.5 to -0.1)	**0.04**	NA		

© 2025 National Cancer Registry Programme Investigator Group. *JAMA Network Open*.

Females (continued).

Registry	All years		Trend 1			Trend 2			Trend 3		
	AAPC, % (95% CI)	p-value	Years	APC, % (95% CI)	p-value	Years	APC, % (95% CI)	p-value	Years	APC, % (95% CI)	p-value
**Wardha**	2.7 (1.0 to 4.4)	**0.01**	2010 - 2019	2.7 (1.0 to 4.4)	**0.01**	NA			NA		
**Ahmedabad Urban**	1.0 (-0.9 to 3.0)	0.29	2008 - 2012	2.2 (-2.5 to 7.2)	0.29	NA			NA		
**Aurangabad**	2.2 (0.8 to 3.5)	**0.004**	2005 - 2019	2.2 (0.8 to 3.5)	**0.004**	NA			NA		
**Barshi rural**	0.1 (-0.6 to 0.8)	0.83	2004 - 2018	0.1 (-0.6 to 0.8)	0.83	NA			NA		
**Mumbai**	0.6 (0.1 to 1.1)	**0.03**	2004 - 2018	0.6 (0.1 to 1.1)	**0.03**	NA			NA		
**Osmanabad & Beed**	-2.3 (-3.2 to -1.4)	**< 0.001**	2008 - 2019	-2.3 (-3.2 to -1.4)	**< 0.001**	NA			NA		
**Pune**	1.8 (0.5 to 3.2)	**0.01**	2006 - 2019	1.8 (0.5 to 3.2)	**0.01**	NA			NA		
**Bangalore**	1.9 (1.3 to 2.4)	**< 0.001**	2004 - 2018	1.9 (1.3 to 2.4)	**< 0.001**	NA			NA		
**Chennai**	1.3 (1.0 to 1.6)	**< 0.001**	2004 - 2018	1.3 (1.0 to 1.6)	**< 0.001**	NA			NA		
**Kollam**	2.4 (1.7 to 3.0)	**< 0.001**	2006 - 2011	-0.1 (-1.6 to 1.4)	0.86	2011 - 2019	3.9 (3.2 to 4.7)	**< 0.001**	NA		
**Thiruvananthapuram Taluk**	3.4 (3.1 to 3.8)	**< 0.001**	2005 - 2019	3.4 (3.1 to 3.8)	**< 0.001**	NA			NA		

Abbreviations: PBCR - Population Based Cancer Registry; AAPC, average annual percent change; APC, annual percent change. Statistical significance at p < 0.05.
Note: All sites - all cancers combined.

© 2025 National Cancer Registry Programme Investigator Group. *JAMA Network Open*.

eFigure 2. AAPC and Trends in Age-Adjusted Incidence Rate (2002-2019) for Selected Sites of Cancer

Males

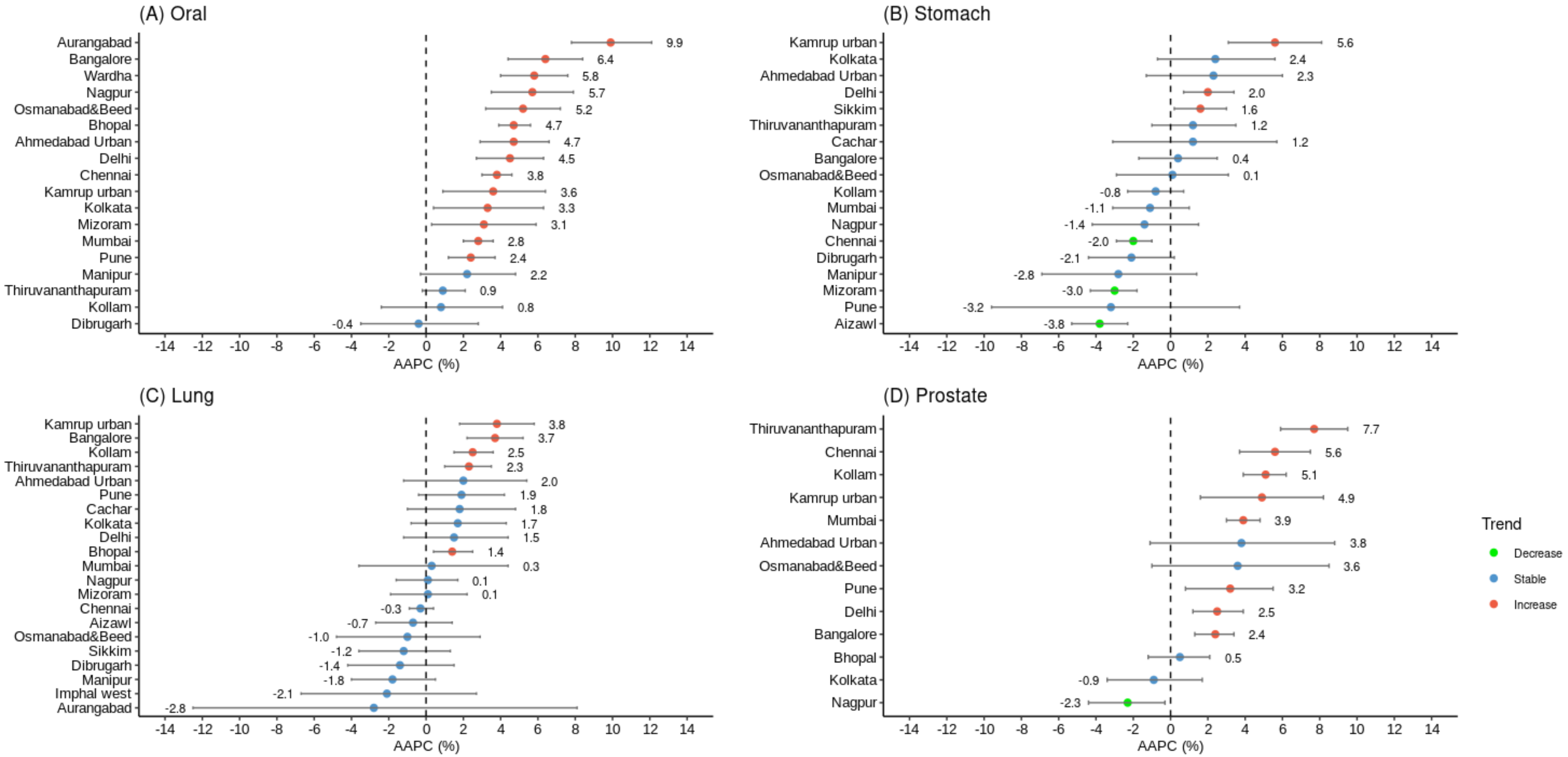

© 2025 National Cancer Registry Programme Investigator Group. *JAMA Network Open*.

Females

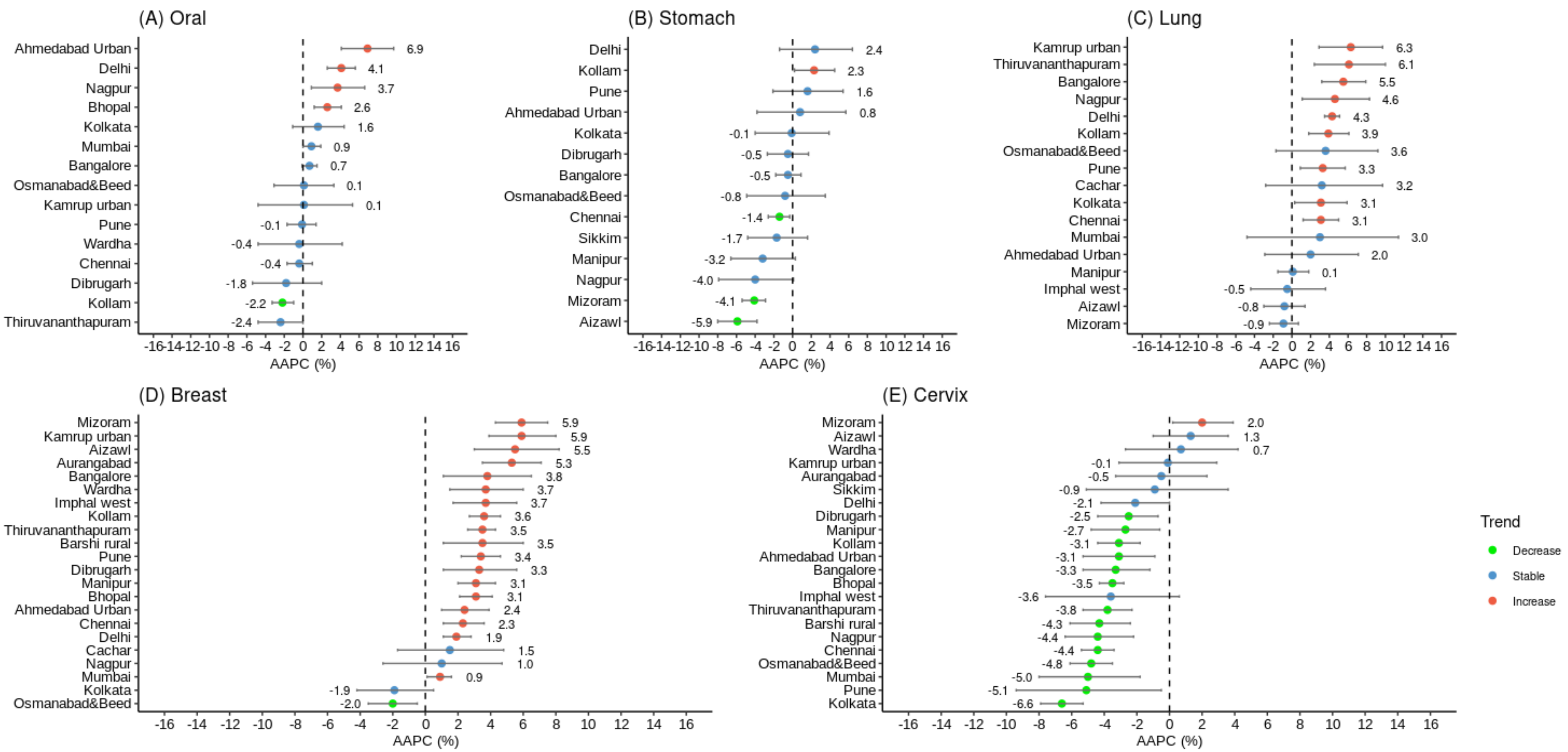

Whiskers indicate 95% Confidence Intervals.
Abbreviation: AAPC- Average Annual percentage change.
Note: Trends in age-adjusted incidence rates across the population were performed based on the World Standard Population (WSP)

© 2025 National Cancer Registry Programme Investigator Group. *JAMA Network Open*.

Note: Year Inclusion - Delhi (2002-2017); Mumbai, Bangalore, Chennai, Kamrup Urban, Dibrugarh - 2004-2018; Bhopal, Barshi Rural, Mizoram, Aizawl, Aurangabad, Nagpur, Thiruvananthapuram taluk - 2005-2019; Sikkim -2005-2018; Kolkata -2006-2017; Manipur, Imphal West, Pune, Kollam -2006-2019; Ahmedabad Urban-2008-2018; Osmanabad & Beed, Cachar -2008-2019; Wardha- 2010-2019; PBCRs with small numbers (< 10 cases) per year not analysed.

eFigure 3. Trends in Age-Adjusted Mortality Rate (2002-2019) for All Sites of Cancer

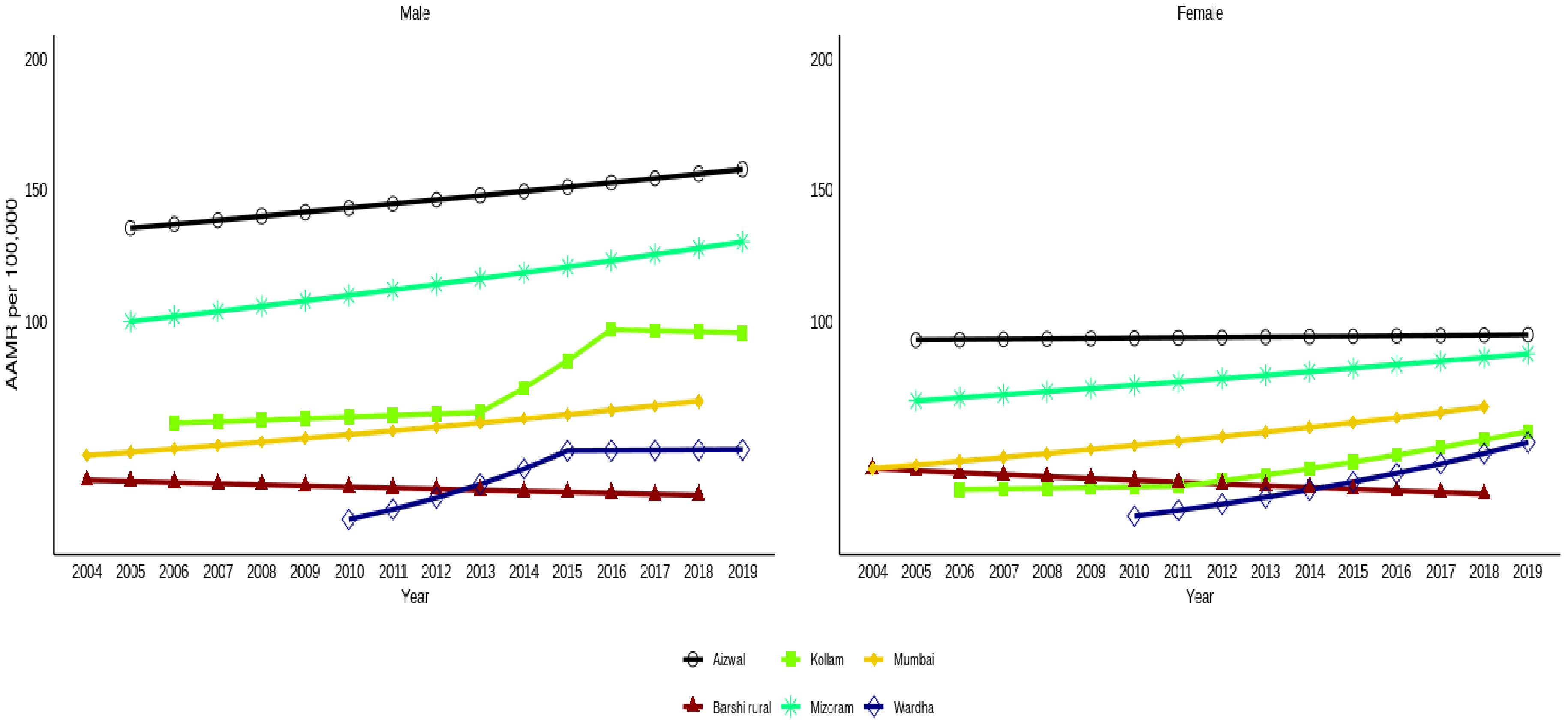

Note: All sites- all cancers combined.

© 2025 National Cancer Registry Programme Investigator Group. *JAMA Network Open*.

eTable 4. AAPC and Trends in Age-Adjusted Mortality Rate (2002-2019) for All Sites of Cancer

Male											
**Registry**	**All years**		**Trend 1**			**Trend 2**			**Trend 3**		
	**AAPC, % (95% CI)**	**p-value**	**Years**	**APC, % (95% CI)**	**p-value**	**Years**	**APC, % (95% CI)**	**p-value**	**Years**	**APC, % (95% CI)**	**p-value**
**Aizawl**	1.1 (-0.2 to 2.5)	0.10	2005-2019	1.1 (-0.2 to 2.5)	0.10	NA			NA		
**Mizoram**	1.9 (0.8 to 3.0)	**0.002**	2005-2019	1.9 (0.8 to 3.0)	**0.002**	NA			NA		
**Mumbai**	2.6 (1.0 to 4.2)	**0.004**	2004-2018	2.6 (1.0 to 4.2)	**0.004**	NA			NA		
**Barshi Rural**	-1.1 (-2.7 to 0.5)	0.16	2004-2018	-1.1 (-2.7 to 0.5)	0.16	NA			NA		
**Kollam**	3.5 (-0.1 to 7.2)	0.06	2006-2013	0.9 (-1.3 to 3.2)	0.34	2013-2016	14.1 (-3.2 to 34.5)	0.10	2016-2019	-0.5 (-8.3 to 8.1)	0.90
**Wardha**	8.5 (3.7 to 13.6)	**<0.001**	2010-2015	15.7 (7.7 to 24.3)	**0.003**	2015-2019	0.2 (-9.5 to 10.9)	0.96	NA		
**Female**											
**Registry**	**All years**		**Trend 1**			**Trend 2**			**Trend 3**		
	**AAPC, % (95% CI)**	**p-value**	**Years**	**APC, % (95% CI)**	**p-value**	**Years**	**APC, % (95% CI)**	**p-value**	**Years**	**APC, % (95% CI)**	**p-value**
**Aizawl**	0.2 (-1.1 to 1.4)	0.80	2005-2019	0.2 (-1.1 to 1.4)	0.80	NA			NA		
**Mizoram**	1.7 (0.3 to 3.0)	**0.02**	2005-2019	1.7 (0.3 to 3.0)	**0.02**	NA			NA		
**Mumbai**	3.1 (1.7 to 4.5)	**<0.001**	2004-2018	3.1 (1.7 to 4.5)	**<0.001**	NA			NA		
**Barshi Rural**	-1.8 (-2.9 to -0.7)	**0.004**	2004-2018	-1.8 (-2.9 to -0.7)	**0.004**	NA			NA		
**Kollam**	3.8 (1.6 to 6.0)	**0.001**	2006-2011	0.7 (-4.4 to 5.9)	0.78	2011-2019	5.8 (3.2 to 8.5)	**0.001**	NA		
**Wardha**	8.6 (5.0 to 12.3)	**<0.001**	2010-2019	8.6 (5.0 to 12.3)	**<0.001**	NA			NA		

Abbreviations: PBCR - Population Based Cancer Registry; AAPC, average annual percent change; APC, annual percent change. Statistical significance at p < 0.05.
Note: PBCRs with an M:I ratio > 0.50 and longer period (10 or more years) of data availability were included in the trend analysis; All sites - all cancers combined.

© 2025 National Cancer Registry Programme Investigator Group. *JAMA Network Open*.

eFigure 4. Top 3 Leading Sites of Cancers Based on the Relative Proportion in PBCRs in India (2015-2019)

	Males 1	Males 2	Males 3	Males TOP3 (%)	Females 1	Females 2	Females 3	Females TOP3 (%)
Kashmir	Stomach (21.2)	Lung (16.0)	Oesophagus (7.3)	44.5	Breast (17.4)	Stomach (9.4)	Oesophagus (8.5)	35.3
Pulwama	Stomach (21.3)	Lung (15.9)	Oesophagus (12.5)	49.7	Breast (16.5)	Oesophagus (12.1)	Stomach (9.2)	37.8
Srinagar	Lung (22.2)	Stomach (16.0)	Prostate (7.9)	46.1	Breast (22.6)	Stomach (10.7)	Colon (8.5)	41.8
Delhi	Lung (10.7)	Mouth (7.9)	Prostate (7.1)	25.7	Breast (27.4)	Cervix Uteri (8.7)	Gallbladder (7.8)	43.9
Gautam Buddha Nagar	Lung (11.9)	Prostate (8.3)	Mouth (8.1)	28.4	Breast (34.0)	Cervix Uteri (7.5)	Ovary (6.9)	48.6
Prayagraj	Mouth (21.1)	Tongue (9.8)	Lung (8.3)	39.2	Breast (22.9)	Gallbladder (16.1)	Cervix Uteri (13.3)	52.3
Varanasi	Mouth (25.3)	Tongue (7.9)	Gallbladder (5.5)	38.7	Breast (22.6)	Gallbladder (13.5)	Cervix Uteri (11.9)	48.0
Patiala	Oesophagus (9.5)	Prostate (7.2)	Mouth (6.7)	23.4	Breast (30.5)	Cervix Uteri (12.6)	Oesophagus (7.3)	50.4
Sangrur	Prostate (9.7)	Liver (9.6)	Oesophagus (7.5)	26.8	Breast (24.2)	Cervix Uteri (11.8)	Oesophagus (7.5)	43.5
Mansa	Prostate (7.8)	Liver (7.0)	Oesophagus (6.6)	21.4	Breast (23.7)	Cervix Uteri (14.6)	Oesophagus (9.9)	48.1
Chandigarh	Lung (11.7)	Prostate (9.6)	Mouth (6.0)	27.3	Breast (30.7)	Cervix Uteri (7.2)	Ovary (7.2)	45.1
SAS Nagar	Prostate (11.1)	Lung (7.6)	Oesophagus (7.5)	26.3	Breast (31.9)	Cervix Uteri (9.2)	Ovary (6.9)	48.1
Bhopal	Mouth	Tongue	Lung	39.8	Breast	Cervix Uteri	Ovary	50.6

© 2025 National Cancer Registry Programme Investigator Group. *JAMA Network Open*.

	(18.4)	(10.8)	(10.6)		(32.2)	(10.6)	(7.8)	
**Ahmedabad Urban**	**Mouth (23.4)**	**Tongue (11.7)**	**Lung (7.9)**	**43.0**	**Breast (33.1)**	**Cervix Uteri (7.9)**	**Mouth (5.7)**	**46.7**
**Muzaffarpur**	**Mouth (16.2)**	**Prostate (6.0)**	**Tongue (5.7)**	**27.9**	**Breast (26.9)**	**Cervix Uteri (15.8)**	**Gallbladder (10.9)**	**53.5**
**Cachar**	**Hypopharynx (12.2)**	**Oesophagus (12.1)**	**Tongue (9.5)**	**33.7**	**Breast (14.5)**	**Cervix Uteri (13.0)**	**Gallbladder (11.6)**	**39.2**
**Karimganj**	**Lung (11.2)**	**Oesophagus (10.9)**	**Hypopharynx (9.7)**	**31.7**	**Gallbladder (16.0)**	**Breast (13.7)**	**Cervix Uteri (10.0)**	**39.7**
Hailakandi	**Lung (11.1)**	**Mouth (9.4)**	**Hypopharynx (8.7)**	**29.2**	**Gallbladder (18.1)**	**Breast (11.5)**	**Cervix Uteri (9.3)**	**38.8**
Dima Hasao	**Lung (17.8)**	**Oesophagus (14.7)**	**Mouth (10.1)**	**42.6**	**Breast (16.9)**	**Ovary (16.9)**	**Cervix Uteri (10.8)**	**44.6**
Karimganj District	**Oesophagus (11.9)**	**Lung (10.9)**	**Hypopharynx (9.9)**	**32.7**	**Gallbladder (16.5)**	**Breast (14.3)**	**Cervix Uteri (10.4)**	**41.2**
**Dibrugarh**	**Oesophagus (16.8)**	**Hypopharynx (11.8)**	**Mouth (7.9)**	**36.5**	**Breast (22.2)**	**Ovary (9.7)**	**Oesophagus (9.1)**	**41.0**
**Kamrup Urban**	**Oesophagus (13.6)**	**Hypopharynx (10.2)**	**Lung (7.7)**	**31.5**	**Breast (18.7)**	**Gallbladder (8.5)**	**Oesophagus (8.1)**	**35.3**
**Tripura**	**Lung (17.7)**	**Oesophagus (7.3)**	**Larynx (6.7)**	**31.6**	**Breast (16.2)**	**Cervix Uteri (15.5)**	**Gallbladder (9.2)**	**41.0**
**Sikkim**	**Stomach (16.2)**	**Oesophagus (9.1)**	**Liver (8.1)**	**33.4**	**Breast (13.0)**	**Cervix Uteri (8.7)**	**Stomach (8.4)**	**30.0**
**Mizoram**	**Stomach (16.4)**	**Oesophagus (16.2)**	**Lung (13.0)**	**45.6**	**Breast (15.6)**	**Cervix Uteri (14.5)**	**Lung (12.0)**	**42.1**
Aizawl	**Oesophagus (18.5)**	**Stomach (14.8)**	**Lung (12.8)**	**46.1**	**Breast (17.4)**	**Cervix Uteri (14.2)**	**Lung (13.8)**	**45.3**
**West Arunachal**	**Stomach (19.0)**	**Liver (18.4)**	**Oesophagus (7.8)**	**45.2**	**Stomach (16.0)**	**Breast (12.9)**	**Cervix Uteri (10.8)**	**39.7**

© 2025 National Cancer Registry Programme Investigator Group. *JAMA Network Open*.

PapumPare	Liver (14.8)	Stomach (14.6)	Lung (8.3)	37.7	Breast (15.9)	Cervix Uteri (11.4)	Stomach (10.4)	37.6
Pasighat	Stomach (18.6)	Lung (9.2)	Oesophagus (7.3)	35.1	Breast (13.8)	Cervix uteri (13.6)	Stomach (9.8)	37.2
Meghalaya	Oesophagus (31.5)	Hypopharynx (9.1)	Larynx (6.4)	47.0	Oesophagus (22.2)	Mouth (9.1)	Cervix Uteri (8.8)	40.1
East Khasi Hills	Oesophagus (35.5)	Hypopharynx (9.1)	Larynx (6.5)	51.1	Oesophagus (26.4)	Breast (9.4)	Mouth (8.0)	43.9
Manipur	Lung (18.5)	Stomach (7.2)	Nasopharynx (6.1)	31.8	Breast (15.7)	Lung (14.0)	Cervix Uteri (9.2)	39.0
Imphal West	Lung (18.9)	Colon (5.6)	NHL (5.3)	29.8	Breast (15.9)	Lung (13.7)	Cervix Uteri (8.6)	38.2
Nagaland	Nasopharynx (12.7)	Oesophagus (10.2)	Stomach (9.9)	32.8	Cervix Uteri (16.7)	Breast (11.6)	Thyroid (8.9)	37.2
Kolkata	Lung (19.5)	Mouth (7.5)	Prostate (7.2)	34.2	Breast (24.4)	Cervix Uteri (7.9)	Gallbladder (7.2)	39.5
Wardha district	Mouth (18.3)	Lung (7.5)	Tongue (7.3)	33.1	Breast (29.2)	Cervix Uteri (10.7)	Ovary (7.3)	47.2
Barshi Rural	Mouth (9.8)	Prostate (8.3)	Liver (7.7)	25.8	Cervix Uteri (22.0)	Breast (21.8)	Ovary (7.6)	51.4
Mumbai	Mouth (10.5)	Prostate (9.1)	Lung (8.7)	28.3	Breast (30.4)	Cervix Uteri (6.4)	Ovary (6.2)	43.0
Aurangabad	Mouth (20.9)	Tongue (11.3)	Lung (8.9)	41.2	Breast (36.6)	Cervix Uteri (15.4)	Ovary (5.4)	57.4
Osmanabad & Beed	Mouth (15.7)	Tongue (10.0)	Prostate (7.0)	32.7	Cervix Uteri (26.3)	Breast (24.2)	Ovary (5.3)	55.7
Pune	Mouth (10.7)	Prostate (9.7)	Lung (7.3)	27.7	Breast (33.6)	Cervix Uteri (8.4)	Ovary (6.3)	48.3
Nagpur	Mouth (20.3)	Tongue (9.3)	Lung (6.4)	35.9	Breast (30.2)	Cervix Uteri (10.9)	Mouth (5.8)	46.9

© 2025 National Cancer Registry Programme Investigator Group. *JAMA Network Open*.

Sindhudurg	Mouth (21.4)	Tongue (8.5)	Oesophagus (8.0)	37.8	Breast (33.3)	Mouth (10.6)	Ovary (7.4)	51.3
Ratnagiri	Mouth (23.3)	Oesophagus (6.4)	Tongue (6.3)	36.0	Breast (25.2)	Mouth (10.3)	Cervix Uteri (9.1)	44.6
Hyderabad	Mouth (13.0)	Lung (10.0)	Tongue (7.8)	30.8	Breast (35.2)	Cervix Uteri (7.9)	Ovary (6.7)	49.7
Visakhapatnam	Lung (10.1)	Mouth (8.8)	Tongue (8.6)	27.5	Breast (25.0)	Cervix Uteri (23.3)	Ovary (6.2)	54.4
Bangalore	Lung (9.9)	Prostate (7.0)	Stomach (6.6)	23.5	Breast (31.6)	Cervix Uteri (9.1)	Ovary (6.4)	47.1
Malabar	Lung (21.0)	Stomach (6.7)	Mouth (5.4)	33.1	Breast (30.0)	Ovary (6.5)	Cervix Uteri (6.0)	42.5
Kannur	Lung (21.8)	Stomach (6.9)	Prostate (5.6)	34.2	Breast (30.0)	Ovary (6.3)	Cervix Uteri (5.4)	41.7
Kasaragod	Lung (19.3)	Mouth (8.5)	Tongue (6.2)	34.0	Breast (30.1)	Cervix Uteri (7.7)	Ovary (7.0)	44.8
Kollam	Lung (17.0)	Prostate (6.3)	Liver (6.1)	29.4	Breast (30.2)	Thyroid (11.0)	Cervix Uteri (5.2)	46.4
Thiruvananthapuram	Lung (12.9)	Prostate (7.9)	Liver (5.5)	26.3	Breast (29.9)	Thyroid (11.0)	Corpus Uteri (5.3)	46.1
Pathanamthitta	Lung (13.8)	Prostate (7.5)	Liver (6.8)	28.1	Breast (28.3)	Thyroid (7.1)	Ovary (7.1)	42.5
Alappuzha	Lung (16.1)	Prostate (6.9)	Liver (4.9)	27.9	Breast (33.3)	Thyroid (6.4)	Ovary (6.0)	45.7
Tamil Nadu	Stomach (8.9)	Mouth (8.2)	Lung (8.1)	25.2	Breast (26.2)	Cervix Uteri (20.0)	Ovary (5.4)	51.6
Chennai	Lung (9.5)	Mouth (9.1)	Stomach (7.9)	26.5	Breast (33.3)	Cervix Uteri (9.3)	Ovary (5.8)	48.4

RANK

© 2025 National Cancer Registry Programme Investigator Group. *JAMA Network Open*.

eTable 5. Cancer Mortality Cases: Number, Mortality-Incidence Ratio (M/I), and Rates (CMR and AAMR) per 100 000 by Sex in 43 PBCRs (2015-2019), India

SN.	PBCR (Ref. Year)	No. of Deaths			CMR		AAMR (WSP)		AAMR (ISP)		No. of New Cases			M/I
		Male	Female	Total	Male	Female	Male	Female	Male	Female	Male	Female	Total	Total
1	Kashmir Province, Jammu & Kashmir (2018-19)	870	558	1428	11.3	8.2	16.6	10.8	12.6	8.5	7113	5570	12683	11.3
	Pulwama, Jammu & Kashmir (2018-19)	79	47	126	15.0	10.0	19.1	11.1	14.7	9.0	694	612	1306	9.6
	Srinagar, Jammu & Kashmir (2018 19)	266	167	433	20.4	13.6	23.5	14.4	17.5	11.1	2033	1628	3661	11.8
2	Delhi, UT (2015-17)	5583	4163	9746	19.0	15.8	23.9	18.2	19.0	14.7	34303	30479	64782	15.0
3	Gautam Buddha Nagar, Uttar Pradesh (2016-18)	354	235	589	10.9	8.4	16.2	11.9	12.4	9.2	2906	2696	5602	10.5
4	Prayagraj, Uttar Pradesh (2017-2019)	993	776	1769	9.3	7.9	11.9	9.9	9.5	7.9	5542	4824	10366	17.1
5	Varanasi, Uttar Pradesh (2018-19)	1554	1076	2630	36.9	27.9	43.6	32.3	35.3	26.1	2619	2013	4632	56.8
6	Muzaffarpur, Bihar (2018)	210	211	421	20.0	22.3	24.2	27.3	19.8	22.3	383	368	751	56.1
7	Patiala district, Punjab (2015-18)	772	774	1546	17.3	19.4	18.1	18.5	14.2	14.7	2960	3387	6347	24.4
8	Sangrur, Punjab (2017-18)	1027	875	1902	53.9	52.0	56.8	51.8	44.7	41.2	1338	1369	2707	70.3
9	Mansa, Punjab (2017-18)	405	416	821	46.0	53.3	51.7	46.5	34.8	36.9	561	617	1178	69.7

© 2025 National Cancer Registry Programme Investigator Group. *JAMA Network Open*.

eTable 5: Cancer Mortality cases: Number, Mortality-Incidence ratio (M/I), Rates (CMR, AAMR) per 100,000 by sex in 43 PBCRs (2015-2019), India. (continued).

SN.	PBCR (Ref. Year)	No. of Deaths			CMR		AAMR (WSP)		AAMR (ISP)		No. of New Cases			M/I
		Male	Female	Total	Male	Female	Male	Female	Male	Female	Male	Female	Total	Total
10	Chandigarh, Punjab (2017-18)	623	485	1108	46.9	43.5	62.3	51.7	47.9	40.1	968	990	1958	56.6
11	SAS Nagar, Punjab (2017-18)	556	419	975	41.9	34.8	53.8	41.9	41.4	32.9	917	1030	1947	50.1
12	Bhopal, Madhya Pradesh (2016-19)	1405	1038	2443	29.8	23.5	34.5	26.5	32.7	26.1	4638	4304	8942	27.3
13	Ahmedabad Urban, Gujarat (2015-18)	4660	2957	7617	33.2	23.3	35.9	23.6	28.8	19.1	13472	10553	24025	31.7
14	Cachar district, Assam (2015-19)	1437	1025	2462	29.0	21.3	38.3	27.1	29.5	21.7	5283	4637	9920	24.8
15	Karimganj[a], Assam (2016-18)	460	352	812	12.7	10.1	17.7	14.0	13.8	11.2	2192	1539	3731	21.8
	Hailakandi, Assam (2016-18)	154	113	267	13.5	10.3	18.4	14.4	14.5	11.7	702	443	1145	23.3
	Dima Hasao, Assam (2016-18)	27	10	37	7.6	2.9	11.4	4.8	8.7	3.7	129	65	194	19.1
	Karimganj district, Assam (2016-18)	277	228	505	13.1	11.1	18.2	15.2	14.1	12.1	1274	946	2220	22.7
16	Dibrugarh district, Assam (2015-18)	602	416	1018	21.0	14.8	26	17.1	13.9	9.1	2073	1909	3982	25.6
17	Kamrup Urban, Assam (2015-18)	1908	1236	3144	69.3	44.9	76.1	50.3	58.1	39.4	5384	4395	9779	32.2
18	Tripura state (2015-18)	3305	2220	5525	40.8	28.3	49.3	31.5	37.8	25.4	6049	4645	10694	51.7

© 2025 National Cancer Registry Programme Investigator Group. *JAMA Network Open*.

eTable 5: Cancer Mortality cases: Number, Mortality-Incidence ratio (M/I), Rates (CMR, AAMR) per 100,000 by sex in 43 PBCRs (2015-2019), India. (continued).

SN.	PBCR (Ref. Year)	No. of Deaths			CMR		AAMR (WSP)		AAMR (ISP)		No. of New Cases			M/I
		Male	Female	Total	Male	Female	Male	Female	Male	Female	Male	Female	Total	Total
19	Sikkim state (2015-18)	492	396	888	35.6	31.9	44.3	40.5	33.7	32.4	1054	994	2048	43.4
20	Mizoram state (2015-19)	2784	1961	4745	88.8	62.4	125.2	87.1	95.7	67.5	4501	4232	8733	54.3
	Aizawl, Mizoram (2015-19)	1345	901	2246	120.6	77.2	155.9	96.4	118.9	74.5	2253	2124	4377	51.3
21	West Arunachal[c], Arunachal Pradesh (2015-19)	344	234	578	14.6	10.1	28.9	19.3	22.3	15.4	1392	1214	2606	22.2
	Papumpare, Arunachal Pradesh (2015-19)	140	98	238	25.5	17.2	66.0	43.4	49.9	33.8	515	473	988	24.1
22	Pasighat*, Arunachal Pradesh (2015-19)	140	104	244	38.6	29.0	54.3	41.5	42.0	32.8	382	369	751	32.5
23	Meghalaya[d], Meghalaya (2015-19)	2978	1751	4729	54.9	31.8	107.4	55.9	84.4	43.7	5414	3352	8766	53.9
	East Khasi Hills, Meghalaya (2015-19)	1821	1134	2955	77.7	47.0	138.3	72.3	108.6	56.2	3269	2042	5311	55.6
24	Manipur state (2015-19)	1763	1761	3524	20.6	20.8	28.5	27.0	21.6	21.0	4166	5017	9183	38.4
	Imphal West, Manipur (2015 – 2019)	525	551	1076	37.7	37.7	41.5	39.1	31.6	30.3	1263	1681	2944	36.5
25	Nagaland[d], Nagaland (2015-19)	339	173	512	16.2	8.8	28.3	13.7	29.9	14.5	1604	1187	2791	18.3
26	Kolkata, West Bengal (2015-17)	3789	3044	6833	55.3	46.6	44	38.1	34.0	29.8	8930	7814	16744	40.8
27	Wardha district, Maharashtra	1987	1765	3752	57.8	53.9	50.6	45.8	40.9	37.1	2714	2860	5574	67.3

© 2025 National Cancer Registry Programme Investigator Group. *JAMA Network Open*.

	(2015-19)												

eTable 5: Cancer Mortality cases: Number, Mortality-Incidence ratio (M/I), Rates (CMR, AAMR) per 100,000 by sex in 43 PBCRs (2015-2019), India. (continued).

SN.	PBCR (Ref. Year)	No. of Deaths			CMR		AAMR (WSP)		AAMR (ISP)		No. of New Cases			M/I
		Male	Female	Total	Male	Female	Male	Female	Male	Female	Male	Female	Total	Total
28	Barshi Rural, Maharashtra (2015-19)	558	528	1086	40.0	42.4	35.4	35.3	28.1	27.7	779	870	1649	65.9
29	Mumbai, Maharashtra (2015-18)	16391	15683	32074	60.5	65.9	64.8	62.6	49.9	48.6	27866	28703	56569	56.7
30	Aurangabad, Maharashtra (2015-19)	344	261	605	9.3	7.5	12.1	8.8	9.6	7.1	2153	2239	4392	13.8
31	Osmanabad & Beed, Maharashtra (2015-19)	1367	1031	2398	11.2	9.3	10.5	8.0	8.1	6.3	4570	5433	10003	24.0
32	Pune, Maharashtra (2015-19)	4648	4748	9396	29.6	33.3	35.8	37.5	27.7	29.1	11314	13011	24325	38.6
33	Nagpur, Maharashtra (2015-19)	1986	1704	3690	28.5	25.0	28.2	23.6	22.9	19.2	6235	6360	12595	29.3
34	Sindhudurg, Maharashtra (2017-18)	316	261	577	39.3	32.8	29.3	23.7	23.8	19.3	402	462	864	66.8
35	Ratnagiri, Maharashtra (2017-18)	520	577	1097	36.7	37.2	29.6	27.8	24.1	23.2	855	1067	1922	57.1
36	Hyderabad district, Telangana (2015-18)	1178	1350	2528	14.4	17.1	17.0	21.3	13.6	16.9	7868	9966	17834	14.2
37	Visakhapatnam, Andhra Pradesh (2017-18)	1292	1430	2722	28.4	31.1	29.9	29.7	23.8	24.2	2149	3137	5286	51.5

© 2025 National Cancer Registry Programme Investigator Group. *JAMA Network Open*.

38	Bangalore, Karnataka (2015-18)	6004	5722	11726	29.2	29.9	36.5	35.1	28.2	27.4	21321	25331	46652	25.1

eTable 5: Cancer Mortality cases: Number, Mortality-Incidence ratio (M/I), Rates (CMR, AAMR) per 100,000 by sex in 43 PBCRs (2015-2019), India. (continued).

SN.	PBCR (Ref. Year)	No. of Deaths			CMR		AAMR (WSP)		AAMR (ISP)		No. of New Cases			M/I
		Male	Female	Total	Male	Female	Male	Female	Male	Female	Male	Female	Total	Total
39	Malabar, Kerala (2015-18)	5575	3707	9282	74.4	43.4	66.0	34.5	50.0	27.0	12941	11277	24218	38.3
	Kannur, Kerala (2015-18)	4006	2684	6690	83.6	48.1	69.1	36.0	52.2	28.1	9387	8282	17669	37.9
	Kasaragod, Kerala (2015-18)	1544	1007	2551	59.1	35.0	60.1	32.0	46.0	25.2	3469	2913	6382	40.0
40	Kollam district, Kerala (2015-19)	7879	5270	13151	126.6	74.1	95.1	52.4	66.4	38.6	11919	11784	23703	55.5
41	Thiruvananthapuram district, Kerala (2015-19)	7632	5822	13454	96.0	66.3	72.9	47.9	56.1	37.4	14966	16529	31495	42.7
	Pathanamthitta[g], Kerala (2019)	808	525	1333	149.7	83.6	86.6	46.4	66.2	36.6	1408	1316	2724	48.9
	Alappuzha[g], Kerala (2019)	1172	740	1912	115.8	65.4	78.6	40.1	59.0	31.0	2360	2187	4547	42.0
42	Tamil Nadu state (2015-17)	14606	13059	27665	12.5	11.2	11.9	10.0	9.5	8.1	88665	109558	198223	14.0
43	Chennai[h], Tamil Nadu (2015-18)	4346	3781	8127	45.1	38.9	43.0	35.7	33.6	28.1	12630	14728	27358	29.7

Abbreviations: PBCR: Population-based cancer registry; CMR: Crude mortality rate; AAMR: Age-adjusted mortality rate; M/I: Mortality to incidence ratio.
Note: Muzaffarpur covered: Motipur, Kanti, Musahari, Sakra, Muraul, and Muzaffarpur Municipal Corporation; [a]Karimganj covered: Karimganj, Hailakandi and Dima Hasao; [b]West Arunachal covered: Tawang, West Kameng, East Kameng, Upper Subansiri, Lower Subansiri, Kurung Kumey, Papumpare and West Siang; [c]Pasighat covered: East Siang and Upper Siang; [d]Meghalaya covered: East Khasi Hills, West Khasi Hills, Jaintia Hills, and Ri Bhoi Districts; [e]Nagaland covered: Kohima and Dimapur districts; Malabar covered: Kasaragod, Mahe, and Kannur; [g] Pathanamthitta and Alappuzha were expanded districts of Thiruvananthapuram PBCR; [h] Chennai is part of the Tamil Nadu State, and also presented as a separate PBCR.

© 2025 National Cancer Registry Programme Investigator Group. *JAMA Network Open*.

eTable 6. Mortality-to-Incidence Ratio of the Top Leading Cancer Sites Across 43 PBCRs (2015-2019)

SN.	REGISTRY	Breast	Cervix	Prostate	Lung		Oral		Stomach	
					**Male**	**Female**	**Male**	**Female**	**Male**	**Female**
1	Kashmir	4.4	14.1	5.2	20.1	17.8	11.5	8.0	16.0	17.6
	Pulwama	1.0	16.7	14.8	19.1	15.0	0.0	25.0	12.2	8.9
	Srinagar	4.6	16.0	4.8	22.0	20.2	13.8	8.3	18.5	21.1
2	Delhi	8.1	8.7	7.5	25.0	25.0	9.9	9.7	16.4	17.8
3	Gautam Buddha Nagar	5.0	4.0	9.5	21.6	13.6	6.5	5.6	14.9	14.3
4	Prayagraj	7.3	5.5	6.5	31.4	31.1	14.8	13.6	21.5	24.8
5	Varanasi	40.9	59.6	46.6	69.2	65.8	53.5	56.5	68.9	78.0
6	Muzaffarpur	31.3	55.2	30.4	90.9	90.0	41.7	50.0	82.4	171.4
7	Patiala	16.4	23.4	10.9	23.7	25.7	23.6	24.3	33.0	44.4
8	Sangrur	47.7	58.4	56.2	93.0	81.6	68.8	100.0	114.3	100.0
9	Mansa	46.6	61.1	56.8	76.9	92.9	90.6	45.5	112.5	100.0
10	Chandigarh	34.9	43.7	38.7	89.4	100.0	66.7	100.0	76.2	20.0
11	SAS Nagar	24.9	42.1	36.3	94.3	86.4	73.9	44.1	64.7	50.0
12	Bhopal	15.4	20.9	22.2	44.9	43.0	27.8	28.4	38.5	23.2
13	Ahmedabad Urban	24.3	40.1	14.2	35.1	28.8	39.1	39.3	32.2	39.6
14	Cachar	19.3	18.9	27.3	46.2	37.7	24.9	21.4	31.8	35.7
15	Karimganj	11.3	13.6	20.0	30.6	30.9	12.8	10.0	21.7	24.4
	Hailakandi	17.6	17.1	28.6	30.8	36.0	14.8	9.7	20.8	25.0
	Dima Hasao	9.1	14.3	0.0	21.7	0.0	10.0	0.0	20.0	0.0
	Karimganj district	10.4	13.1	16.7	33.1	33.3	11.7	10.9	25.9	28.0
16	Dibrugarh	27.8	29.6	34.2	24.8	15.3	31.7	31.1	27.1	27.2
17	Kamrup Urban	25.8	39.5	25.6	40.8	31.4	35.7	31.6	31.0	32.3
18	Tripura	38.3	41.8	42.4	64.5	64.9	52.4	48.0	61.7	63.0
19	Sikkim	32.6	32.6	46.2	62.7	52.3	23.4	28.6	52.0	50.6
20	Mizoram	23.1	34.3	39.6	72.8	70.3	60.9	37.6	62.6	56.0
	Aizawl	19.5	28.9	37.7	75.4	68.9	55.0	39.5	58.9	53.3
21	West Arunachal	17.9	28.2	10.5	32.0	19.4	37.5	24.0	21.9	24.7
	Papumpare	18.7	31.5	10.0	34.9	33.3	43.5	50.0	29.3	18.4
22	Pasighat	25.5	40.0	23.1	28.6	31.8	32.0	8.3	42.3	38.9

© 2025 National Cancer Registry Programme Investigator Group. *JAMA Network Open*.

eTable 6: Mortality-to-Incidence ratio of the top leading cancer sites across 43 PBCRs (2015-2019) (Continued).

SN.	REGISTRY	Breast	Cervix	Prostate	Lung		Oral		Stomach	
					Male	Female	Male	Female	Male	Female
23	Meghalaya	48.1	51.0	30.8	66.1	69.4	58.3	57.0	50.5	59.7
	East Khasi hills	46.9	45.6	31.0	72.1	72.7	58.1	56.0	52.7	66.9
24	Manipur	29.9	41.0	32.5	61.2	61.0	45.7	46.5	43.2	35.8
	Imphal West	26.6	34.7	34.4	61.1	55.8	49.3	32.1	49.1	43.5
25	Nagaland	17.4	14.1	11.5	18.4	20.4	19.6	10.6	22.8	12.5
26	Kolkata	30.1	30.8	38.8	56.5	54.7	31.3	34.0	57.1	58.6
27	Wardha	44.7	80.5	59.2	83.7	83.3	68.1	76.5	86.8	83.7
28	Barshi Rural	41.6	68.6	53.8	86.7	83.9	54.0	67.5	80.0	52.0
29	Mumbai	43.2	54.2	47.1	76.2	79.0	50.7	57.7	70.9	71.8
30	Aurangabad	7.2	11.0	24.2	36.5	40.4	8.1	12.3	21.1	16.7
31	Osmanabad & Beed	11.3	14.7	35.4	51.4	45.0	12.7	19.1	27.1	30.1
32	Pune	25.4	31.0	32.7	55.7	59.7	34.4	37.6	53.2	55.7
33	Nagpur	13.0	17.2	16.4	30.5	24.5	22.6	23.4	30.3	33.3
34	Sindhudurg	33.8	80.6	50.0	76.0	100.0	65.0	57.6	133.3	200.0
35	Ratnagiri	42.0	57.7	57.1	66.7	61.5	51.0	43.0	78.7	73.9
36	Hyderabad	14.0	18.6	3.5	14.0	14.7	25.3	27.9	12.3	10.4
37	Visakhapatnam	32.7	40.6	28.9	76.9	59.6	55.6	54.3	84.9	79.2
38	Bangalore	14.7	17.4	14.8	34.4	37.1	18.7	21.2	31.0	36.3
39	Malabar	22.0	31.4	30.8	53.0	45.4	36.0	45.3	52.4	56.1
	Kannur	21.2	31.7	30.3	53.8	45.2	34.6	47.8	54.0	54.8
	Kasaragod	24.5	30.5	32.7	50.8	44.7	38.0	41.1	48.8	61.0
40	Kollam	31.7	57.6	47.1	83.7	75.6	61.6	76.0	76.8	73.7
41	Thiruvananthapuram	25.2	40.9	28.3	66.9	57.9	48.1	44.9	53.4	53.5
	Pathanamthitta	22.5	37.5	31.4	70.1	47.4	56.7	50.0	68.5	76.2
	Alappuzha	19.1	38.1	26.2	55.4	47.8	56.8	44.3	64.0	66.7
42	Tamil Nadu	6.0	7.2	8.1	16.5	16.1	12.5	10.8	15.0	13.9
43	Chennai	15.3	20.3	22.8	44.6	39.1	31.9	23.1	42.8	40.0

© 2025 National Cancer Registry Programme Investigator Group. *JAMA Network Open*.

eTable 7. Data Quality Indicators: Number and Relative Proportion, All Sites of Cancer (2015-2019), Both Sexes

Registry	MV%	DCO%	O&U%	Age Unknown (%)
Kashmir	99.4	0.0	0.5	0.0
Pulwama	99.4	0.0	0.4	0.0
Srinagar	99.4	0.0	0.4	0.0
Ahmedabad urban	98.9	0.0	4.8	0.2
Visakhapatnam	97.9	0.5	2.7	0.0
Aurangabad	97.7	0.7	2.6	0.0
SAS Nagar	97.5	0.1	4.1	0.0
Nagaland	96.8	0.0	2.4	0.0
Bhopal	96.5	1.2	2.6	0.0
Hyderabad	95.9	0.3	1.6	0.0
West Arunachal	95.5	0.3	1.8	0.0
Papumpare	95.4	0.3	2.1	0.0
Chandigarh	95.0	0.6	2.5	0.0
Manipur	93.9	1.1	2.7	0.0
Imphal West	93.2	2.0	3.3	0.0
Tripura	92.6	0.8	5.8	0.0
Sindhudurg	92.0	2.3	4.9	0.0
Prayagraj	91.2	0.4	0.7	0.0
Kannur	91.0	2.9	3.6	0.1
Malabar	90.9	3.1	3.7	0.1
Bangalore	90.7	6.1	6.5	0.0
Pune	90.5	3.5	4.7	0.0
Kasaragod	90.3	3.7	3.9	0.1
Gautam Buddha Nagar	89.7	4.0	1.1	0.0
Ratnagiri	89.5	0.5	5.9	0.0
Sikkim	89.2	4.4	8.3	0.0
Osmanabad & Beed	88.1	0.1	8.4	0.7
East Khasi Hills	88.0	6.6	4.7	0.0

© 2025 National Cancer Registry Programme Investigator Group. *JAMA Network Open*.

eTable 7. Data Quality Indicators - Number (n), and Relative Proportion (%), All sites of cancer (2015-2019) – Both males and Females. (Continued)

**Registry**	**MV%**	**DCO%**	**O&U%**	**Age Unknown (%)**
Wardha	87.7	7.0	1.7	0.0
Aizawl	87.7	1.9	5.4	0.1
Mumbai	87.5	8.1	5.0	0.0
Delhi	87.4	4.0	5.0	0.7
Kolkata	87.2	7.9	4.5	0.0
Meghalaya	86.4	8.2	6.4	0.1
Tamil Nadu	86.1	4.4	7.0	0.0
Patiala	85.8	10.6	8.3	0.1
Nagpur	85.4	6.0	13.3	0.0
Mizoram	85.0	4.0	8.0	0.1
Pasighat	84.8	0.7	2.8	0.0
Pathanamthitta	84.8	8.4	9.5	0.0
Barshi Rural	84.7	0.5	5.5	0.0
Hailakandi	84.4	0.3	11.5	0.0
Kamrup Urban	83.4	7.6	5.0	0.0
Alappuzha	82.7	8.4	9.3	0.0
Thiruvananthapuram	82.5	7.3	8.7	0.0
Cachar	82.5	3.5	4.3	0.0
Kollam	82.1	5.0	6.6	0.1
Karimganj district	81.8	0.1	8.5	0.1
Karimganj	81.5	0.3	9.2	0.1
Chennai	80.0	7.4	5.6	0.0
Dibrugarh	79.4	7.8	3.9	0.0
Sangrur	79.0	3.8	6.6	0.0
Muzaffarpur	77.0	1.5	12.3	0.0
Mansa	72.4	8.7	9.8	0.0
Dima Hasao	69.6	2.6	7.7	0.0
Varanasi	65.7	0.5	5.7	0.0

Abbreviations: MV, Microscopic verification; DCO, Death certification only; O &U, Others and unspecified. (O & U – ICD 10: C26, C39, C48, C75, C76, C77, C78, C79, C80, C97).

© 2025 National Cancer Registry Programme Investigator Group. *JAMA Network Open*.
