# Supplementary material for: Cancer Incidence and Mortality Across 43 Cancer Registries in India
Source: JAMA Netw Open. 2025 Aug 20;8(8):e2527805. doi: 10.1001/jamanetworkopen.2025.27805 (PMC12368690; doi:10.1001/jamanetworkopen.2025.27805)
Supplement: Supplement 2. — Data Sharing Statement [file jamanetwopen-e2527805-s002.pdf]

Data Sharing Statement

Mathur. Patterns and Trends in Cancer Incidence and Mortality Across 43 Cancer Registries in India. *JAMA Netw Open*. Published August 20, 2025.
doi:10.1001/jamanetworkopen.2025.27805

Data
Data available: Yes
Data types: Deidentified participant data
How to access data: De-identified data collected for this study are available upon a reasonable request over email to the corresponding author after approval of a proposal along with a signed data access agreement. Also interactive website will be developed to provide cancer statistics after publication.
When available: With publication

Supporting Documents
Document types: None

Additional Information
Who can access the data: Researchers whose proposed use of the data has been approved
Types of analyses: Incidence and mortality rate by age, sex, period and place.
Mechanisms of data availability: After approval of a proposal
